# Diterpenoids and Triterpenoids From Frankincense Are Excellent Anti-psoriatic Agents: An *in silico* Approach

**DOI:** 10.3389/fchem.2020.00486

**Published:** 2020-06-25

**Authors:** Sobia Ahsan Halim, Ajmal Khan, Rene Csuk, Ahmed Al-Rawahi, Ahmed Al-Harrasi

**Affiliations:** ^1^Natural and Medical Sciences Research Center, University of Nizwa, Nizwa, Oman; ^2^Organic Chemistry, Martin-Luther University Halle-Wittenberg, Halle (Saale), Germany

**Keywords:** psoriasis, frankincense, diterpenoid, boswellic acids, *in silico* target fishing, molecular docking

## Abstract

Psoriasis is a chronic autoimmune disease that affects 2–3% of the global population and requires an effective treatment. Frankincense has been long known for its potent anti-inflammatory activities. In this study, a structural bioinformatics approach was used to evaluate the efficacy of individual active components of frankincense, macrocyclic diterpenoid derivatives (**1**-**27**), and boswellic acids (**28**-**46**) in the treatment of psoriasis. Initially, major druggable targets of psoriasis were identified. Subsequently, structure-based screening was employed by using three different docking algorithms and scoring functions (MOE, AutoDock Vina, and MVD) for the target fishing of compounds against 18 possible targets of psoriasis. Janus Kinase 1, 2, 3 (JAK 1/2/3), eNOS, iNOS, interleukin-17 (IL-17), and Tumor necrosis factor-α (TNF-α) were identified as the preferred molecular targets for these compounds. This computational analysis reflects that frankincense diterpenoids and triterpenoids can serve as excellent anti-psoriatic agents by targeting major cytokines (TNF-α, IL-17, IL-13, IL-23, and IL-36γ,) exacerbated in psoriasis, and inflammatory pathways particularly JAK1/2/3, eNOS, iNOS, MAPK2, and IFNγ. The results were compared with the reported experimental findings which correlates well with our *in-silico* verdicts.

## Introduction

Psoriasis is a chronic and most inexplicable autoimmune skin disease that affect 2–3% of the population worldwide (Baliwag et al., 2015). It is characterized by increased propagation of the epidermis that can multiply up to 10 times faster than normal in psoriasis with dilation of dermal capillaries. As underlying cells reach the skin's surface and die, their sheer volume creates red plaques covered with white scales that cause itchy and scaly skin, swelling, pain, and disfiguring skin lesions (MacDonald and Burden, 2007). Psoriasis can occur at any age and equally in men and women. The disease is more common in adults than children. The estimates in children vary between 0.7% (Augustin et al., 2010) in Europe to almost none in Asia (Bø et al., 2008; Chen et al., 2008). The variation in the prevalence of psoriasis has also been linked to geographical locations, as it is less common in countries closer to the Equator (Egypt, Sri-Lanka, Taiwan) as opposed to countries that are further away (Europe, Australia, and North America) (Parisi et al., 2013). Findings have consistently reported an increasing trend of the prevalence of psoriasis (Icen et al., 2009; Egeberg et al., 2017; Eder et al., 2019).

Several factors are involved in causing psoriasis, such as bacterial infection, genetic/environmental factors, and autoimmune disorders. Psoriasis is associated with several comorbidities, including cardiovascular disease (CVD), lymphoma, and extensive depression (Ni and Chiu, 2014; Takeshita et al., 2017). It happens through chronic interactions between hyper-proliferative keratinocytes and infiltrating, activated immune cells. The immune system plays a critical role in the pathogenesis of psoriasis. T cells (particularly Th1 and Th17) are heavily present in psoriatic lesions. Moreover, TNFα and iNOS producing inflammatory dendritic cells, massively infiltrate psoriatic skin, and polarize T cells to Th1 and Th17 fates. Additionally, psoriatic skin is infiltrated by macrophages, innate immune cells, and an increased number of endothelial cells, which exacerbate the pathogenesis of psoriasis. The genetics behind psoriasis is complex and multifactorial. *PSORS* (psoriasis-**s**usceptibility) loci harbor several genes that are involve in psoriasis; for example, *HLA-Cw6, ERAP1, ERAP2*, and *MICA* are involved in antigen presentation. Furthermore, several other genes span an array of functions, i.e., T-cell development and polarization (*RUNX1, RUNX3, STAT3, TAGAP, IL4*, and *IL13*), development of innate immunity (*CARD14, c-REL, TRAF3IP2, DDX58*, and *IFIH1*), the IL-23 axis (*IL12Bp40, IL23Ap19, IL23R, JAK2*, and *TYK2*), and negative regulators of immune responses (*TNIP1, TNFAIP3, NFKBIA, ZC3H12C, IL36RN*, and *SOCS1*) (Al Robaee, 2010; Harden et al., 2015; Woo et al., 2017).

Currently, there are three different types of treatment used to reduce the inflammation and skin irritation/itching, including topical treatments, light therapy, and systemic medications (Winterfield et al., 2005; Gisondi et al., 2017; Golbari et al., 2018). Topical treatments serve as first-line therapies that include use of topical corticosteroids, vitamin D analogs, anthralin, retinoids, and calcineurin inhibitors, and they are frequently prescribed to treat mild to moderate psoriasis. The overuse of corticosteroids causes thinning of the skin. Vitamin D analogs (Calcipotriene, Calcitriol) and anthralin reduce skin cell growth, remove scales, and make skin smoother. These analogs treat mild to moderate psoriasis along with other treatments; however, they promote skin irritation. Similarly, topical retinoids may decrease inflammation, but cause skin irritation and increase sensitivity to sunlight. Moreover, oral retinoids cause risk of birth defects and are not recommended for pregnant and breast-feeding women. Calcineurin inhibitors, particularly tacrolimus and pimecrolimus, also reduce inflammation and plaque accumulation; however, an increased risk of skin cancer and lymphoma is associated with these inhibitors, and they are therefore not recommended for long-term or continuous use (Choi et al., 2017).

Second- or third-line therapies, including phototherapy and systemic therapies, are given to patients with severe psoriasis or treatment-resistant disease. In phototherapy, natural or artificial ultraviolet (UV) light is used to treat mild psoriasis by exposing skin to controlled amounts of natural sunlight, artificial ultraviolet A (UVA), or UV B (UVB) light, either alone or in combination with medications. Exposure to UV in sunlight or artificial light slows skin cell turnover and reduces scaling and inflammation. Daily exposure to small amounts of sunlight may improve psoriasis, but intense sun exposure can worsen symptoms and cause skin damage. Controlled doses of UVB light improves the symptoms of mild to moderate psoriasis; however, it may cause short-term side effects like redness, itching, and dry skin. UVA light penetrates deeper into the skin than UVB, improves skin, and is often used to treat severe psoriasis. Despite this, it can cause short-term side effects, such as nausea, headaches, burning, and itching, or long-term side effects, such as dry/wrinkled skin, freckles, increased sun sensitivity, and increased risk of skin cancer and melanoma (Pardasani et al., 2000).

Patients with severe psoriasis are treated with systemic treatment (including retinoids, methotrexate, and cyclosporine), which are associated with severe side effects. Retinoids may cause lip inflammation and hair loss. Methotrexate helps psoriasis by decreasing the production of skin cells and suppressing inflammation; however, it may also cause stomach upset, loss of appetite, and fatigue. With long-term use, methotrexate can cause liver damage and decreased production of red and white blood cells and platelets. Cyclosporine suppresses the immune system and is similar to methotrexate in effectiveness but can only be taken short term because it may increase risk of infection, cancer, kidney problems, and high blood pressure at high doses or long-term therapy (Hoffman et al., 2016).

The presence of cytokines, dendritic cells, and T lymphocytes in psoriatic lesions has encouraged the development of biologic therapies for psoriasis (Schadler et al., 2019). These therapies include monoclonal antibodies (mAB) against tumor necrosis factor—α (TNF-α) (infliximab, adalimumab, golimumab), interleukin (IL)-12 and IL-23 (ustekinumab), IL-17A (secukinumab and ixekizumab), and inhibitors of TNF-α (etanercept) and phosphodiesterase 4 (apremilast). These drugs are usually used to treat psoriatic patients who have failed to respond to traditional therapy or are associated with psoriatic arthritis. However, these drugs have strong effects on the immune system and may permit life-threatening infections. In particular, people taking these treatments must be screened for tuberculosis. All these treatments are only used to manage the disease at each time it surfaces (Tollefson et al., 2018). Therefore, new and safer chemical agents are urgently required for the effective treatment of psoriasis.

Frankincense is known for its superior anti-inflammatory potential (Hussain et al., 2017; Al-Harrasi et al., 2018a). The active constituents of frankincense, including incensole and several boswellic acid derivatives, suppress the expression of tumor necrosis factor-α (TNF-α), interleukin-1β (IL-1β), and nuclear factor-κβ (NF-κβ) (Moussaieff et al., 2007). Due to our deep interest in exploring medicinal properties of frankincense, a computational pipeline was created to investigate the anti-psoriatic potential of the active components of frankincense. In this study, computational target fishing was applied for the identification of potential druggable molecular targets of psoriasis and the binding potential of cembrenoid diterpenoids and triterpenoids found in several species of *Boswellia* was scrutinized by *in silico* reverse molecular docking. The computational analyses reveal the promising binding potential of these compounds with the proteins associated with psoriatic pathways.

## Materials and Methods

The computational experiments were performed on a Windows 10 workstation with Intel® Core™ i7-7700HQ CPU@2.80GHz processor and 12 GB RAM. For docking, MOE (Molecular Operating Environment), MVD (Molegro Virtual Docker) and ADT Vina (AutoDock Tools Vina) were used. Protein–ligand interactions were visualized on Chimera software (Pettersen et al., 2004).

### Identification of Druggable Proteins in Psoriasis

The major biological pathways of psoriasis were deduced by a literature survey (Rácz and Prens, 2009; Bejarano and Valdecantos, 2013; Baliwag et al., 2015; Hugh and Weinberg, 2018; Yadav et al., 2019), and this revealed that Tumor necrosis factor-α (TNF-α), interleukin-1α (IL-1α), IL-1β, IL-13, IL-12/23, IL-17, IL-22, IL-36γ, Interferon-γ (IFN-γ), Nuclear Factor-κB (NF-κB), endothelial nitric oxide synthase (eNOS), inducible NOS (iNOS), Peroxisome proliferator-activated receptor gamma (PPAR-γ), MAP Kinase-Activated Protein Kinase 2 (MAPK2), Janus Kinase 1 (JAK1), JAK2, JAK3, and the Signal transducer and activator of transcription 3 (STAT3) play important roles in the pathogenesis of psoriasis. Moreover, the druggable macromolecules were also confirmed by Kyoto Encyclopedia of Genes and Genomes (KEGG) database (https://www.kegg.jp/), which showed that the NF-κB, IL-17, and IL-36 pathways are particularly involved in psoriasis (KEGG ID: H01656). Thus, the aforementioned 18 proteins were selected as potential drug targets in our docking studies. The three-dimensional (3D-) coordinates of selected targets were retrieved from the RCSB Protein Data Bank (PDB, https://www.rcsb.org/) with good resolution. The binding site of each protein was elucidated by visual analysis by UCSF chimera (Pettersen et al., 2004) (https://www.cgl.ucsf.edu/chimera/), a literature review, and the PDBsum database (Laskowski et al., 1997) (http://www.ebi.ac.uk/thornton-srv/databases/cgi-bin/pdbsum). For docking, each protein was treated individually. The standard protonation state of each protein was set according to neutral pH, and partial charges were applied based on the MMFF94x force field by MOEv2014.09. All the heteroatoms were deleted from protein structures. By careful analysis, only those water molecules were retained in the protein structures which are involved in protein-ligand bridging, while the rest of the water molecules were removed. The PDB codes of selected targets, their resolution, and their binding residues are tabulated in Table 1. The positive controls were added in the dataset as a reference ligand to test the screening accuracy of docking programs. For IL-17, IL-36γ, PPAR-γ, MAPK2, JAK1/2/3, TNF-α, iNOS, and eNOS, the co-crystallized ligands were chosen as a positive control. The known inhibitors of IFN-γ, IL-22, IL-12/23, IL-1α, IL-1β, NF-κB, STAT3, and IL-13 were selected from https://www.medchemexpress.com/.

**Table 1:** Drug targets used in docking experiments.

**S. no**.	**Molecular targets**	**PDB ID**	**Resolution (Å)**	**Positive control**	**Binding residues**
1	TNF-α	2AZ5	2.1	**^†^6,7-dimethyl-3-[(methyl{2-[methyl({1-[3-(trifluoromethyl)phenyl]-1h-indol-3-yl}methyl)amino]ethyl}amino)methyl]-4h-chromen-4-one**	Leu57(A/B), Tyr59(A/B), Ser60(A/B), Gln61(A), Tyr119(A/B), Leu120(A/B), Gly121(A/B), Gly122(A), Tyr151(A/B).
2	IL-1α	5UC6	2.1	^*^IX207-887, RP54745	Arg16, Lys60, Asp64, Asp65, Ala66, Lys67, Ile68, Trp113, Ile118
3	IL-1β	1ITB	2.5	^*^Byakandelicol, Diacerein	Arg11, Ser13, Gln14, Gln15, Met20, Ser21, Gly22, Lys27, Leu29, His30, Leu31, Gln32, Gly33, Gln34, Asp35, Met36, Gln38, Gln126, Ala127, Glu128, Asn129, Met130, Pro131, Thr147, Gln149
4	IL-13	3LB6	3.05	^*^Suplatast (Tosilate)	Arg11, Ile14, Glu15, Leu101, Lys104, Lys105, Phe107, Arg108, Met33, Trp35, Asp87, Thr88, Lys89, Ile90, Glu91
5	IL-17	5HI5	1.8	**^†^(4S,20R)-7-chloro-N-methyl-4-{[(1-methyl-1H-pyrazol-5-yl)carbonyl]amino}-3,18-dioxo-2,19-diazatetracyclo[20.2.2.1~6,10~.1~11,15~]octacosa-1(24),6(28),7,9,11(27),12,14,22,25-nonaene-20-carboxamide**	Asn36, Pro37, Tyr62, Pro63, Val65, Ile66, Trp67, Gln94, Glu95, Ile96, Leu97, Val98, Leu99, Leu112
6	IL-22	3DLQ	1.9	^*^GSK2981278	Phe47, Gln49, Thr53, Ser64, Asp67, Thr70, Asp71, Arg73, Lys162, Gly165, Glu166, Asp168, Arg175
7	IL-12/23	5MZV	2.8	^*^Apilimod, Isomucronulatol, Tyrphostin A1	Leu56, Arg57, Glu58, Trp156, Leu160, Lys164
8	IL-36γ	6P9E	2.0	**^†^(2S)-2-{[4-(3-amino-4-methylphenyl)-6-methylpyrimidin-2-yl]oxy}-3-methoxy-3,3-diphenylpropanoic acid**	Arg121, Lys123, Val58, Leu130, Leu165, Ile27
9	IFN-γ	1FG9	2.9	^*^AX-024, Pralnacasan	Gln1, Glu9, Lys12, Gly18, Ser20, Ala23, Asp24, Asn25, Gly26, Lys108, His111, Glu112, Gln115, Ala118
10	NF-κB	1A3Q	2.1	^*^(-)-DHMEQ, Daxanabinol	Monomer 1: Arg52, Arg54, Tyr55, Gly56, Cys57, Glu58, His62, Lys143, Lys221, Ser222, Lys252, Gln284 Monomer II: Gly50, Tyr55, Cys57, Lys143, Ser220, Asn227, Lys252, Lys255, Val281, Gln284, Wat1-13
11	eNOS	4D1P	1.731	**^†^6-((((3S, 5R)-5-(((6-amino-4-methylpyridin-2-yl)methoxy)methyl)pyrrolidin-3-yl)oxy)methyl)-4-methylpyridin-2-amine**	Val336, Trp356, Glu361, Phe105, Phe353, Trp447, Trp74, Wat2049, 2282, 2374, 2283, 2372, 2370, 2270, 2279, 2373, 2349 Heme binding: Trp178, Arg183, Val185, Gly186, Cys184, Tyr475, Ser226, Phe353, Ser354, Trp356, Met358, Glu361, Trp447, Phe473, Tyr475
12	iNOS	3E7G	2.2	**^†^Ethyl 4-[(4-methylpyridin-2-yl)amino]piperidine-1-carboxylate**	Ligand binding site: Tyr347, Glu377, Gln263, Arg266, Pro350, Val352, Phe369, Gly371, Trp372, Tyr373, Asp382, Arg388 Heme binding: Cys200, Tyr491, Trp194, Ala197, Gly202, Ser242, Phe369, Asn370, Trp372, Glu377, Trp463, Tyr489, Tyr491, Wat4015, 4048, 4023, 4116, 4121, 4123, 4072
13	PPAR-γ	1NYX	2.65	**^†^(2s)-2-ethoxy-3-{4-[2-(10h-phenoxazin-10-yl)ethoxy]phenyl}propanoic acid**	His323, His449, Tyr473, Ile281, Phe282, Cys285, Gln286, Arg288, Ser289, Ile326, Leu330, Leu240, Ile341, Ser342, Leu453, Leu469, Wat14
14	MAPK2	3KC3	2.9	^†^N4-(7-(benzofuran-2-yl)-1H-indazol-5-yl)pyrimidine-2,4-diamine	Lys93, Leu141, Thr206, Leu70, Gly73, Val78, Ala91, His108, Met138, Glu139, Cys140, Leu141, Asp142, Leu193, Asp207
15	JAK 1	6N7B	1.81	**^†^N-[3-(5-chloro-2-methoxyphenyl)-1-methyl-1H-pyrazol-4-yl]-1H-pyrazolo[4,3-c]pyridine-7-carboxamide**	Leu881, Gly882, Glu883, Gly884, Gly887, Lys888, Val889, Ala906, Met956, Glu957, Phe958, Leu959, Gly962, Glu966, Arg1007, Asn1008, Leu1010, Gly1020, Asp1021, Wat1303, 1351, 1397
16	JAK 2	4BBE	1.9	**^†^N-[4-[2-[(4-morpholin-4-ylphenyl)amino]pyrimidin-4-yl]phenyl]ethanamide**	Lys882, Leu932, Leu855, PHE 860, VAL 863, LYS 882, MET 929, GLU 930, Tyr931, Leu932, Gly935, Leu983, Asp994, Gln853
17	JAK 3	6AAK	2.67	**^†^4-[[(1S,3R)-5-oxidanyl-2-adamantyl]amino]-1H-pyrrolo[2,3-b]pyridine-5-carboxamide**	Leu905, Glu903, Leu828, Ala853, Val884, Met902, Tyr904, Leu905, Gly908, Arg953, Asn954, Leu956, Wat1302, 1309
18	STAT3	6QHD	2.85	^*^STAT3-IN-3, STAT2-IN-1	Arg382(A/B),Ser465(A/B), Asn466(A/B),His332(A/B), Lys340(A/B), Ile467(A/B), Arg417A, Gln469A, Wat1101C, 1102C, 1103C, 1101D, 1103D, 815B, 818A, 821B, 808A

*TNF-α, Tumor necrosis factor-α; IL-1α, Interleukin-1α; IL-1β, Interleukin-1β; IL-13, Interleukin-13; IL-17, Interleukin-17; IL-22, Interleukin-22; IL-23, Interleukin-23; IL-36γ, Interleukin-36 gamma; IFN-γ, Interferon-Gamma; NF-κB, nuclear factor kappa-light-chain-enhancer of activated B cells; eNOS, endothelial Nitric oxide synthase; iNOS, inducible Nitric oxide synthase; PPAR-γ, Peroxisome proliferator-activated receptors-γ; MAPK, Mitogen activated protein Kinase; JAK, Janus Kinase; STAT3, Signal transducer and activator of transcription 3*.

† *These ligands were taken from Protein databank (www.rcsb.org)*.

* *These ligands were taken from MedChemExpress (https://www.medchemexpress.com/)*.

### Selection and Preparation of Ligands for Docking

A set of 46 macrocyclic diterpenoids and triterpenes from different species of *Boswellia* was chosen from our *in-house* compound's library for reverse docking analysis. The structures of the selected compounds are shown in Table 2. The 2D-structure of each ligand was drawn on ChemDraw (https://www.perkinelmer.com/category/chemdraw) and converted into a 3D-format by Molecular Operating Environment (MOE 2014.09) (Florence et al., 2014) (https://www.chemcomp.com/). Hydrogen atoms were added, partial charges were applied (based on MMFF94x force field) on each structure, and the structures were minimized using the MMFF94x force field (eps = r, Cutoff until the RMS gradient of 0.1 kcal/mol/Å was achieved). The protonation state of each compound was assigned based on the neutral pH. All the compounds were imported into MOE database to be used in docking.

**Table 2:** Chemical structures of compounds **1–46**.

**Compounds**	**Name**	**Source**	**Structure**	**References**
**1**	Serratol	*Boswellia serrata*	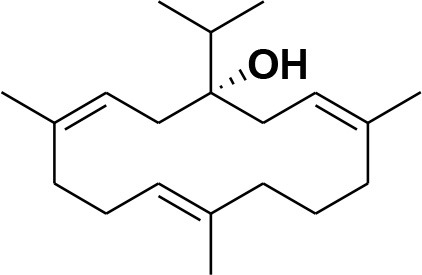	**Schmidt et al.**, **2011**
**2**	Incensole	*Boswellia carterii*	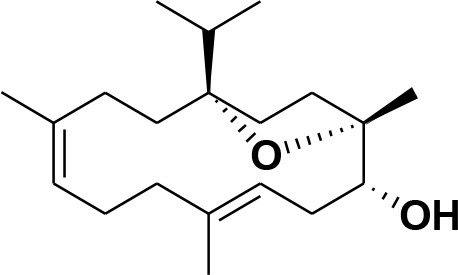	**Corsano and Nicoletti**, **1967**
**3**	Incensole acetate	*Boswellia carterii*	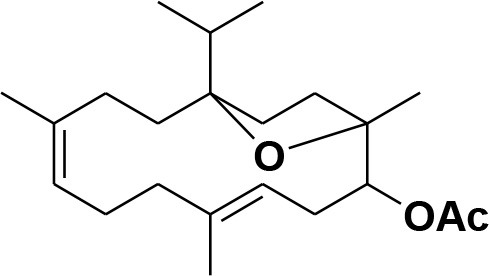	**Obermann**, **1977****; Moussaieff et al.**, **2007**
**4**	Cembrene A	*Boswellia carterii*	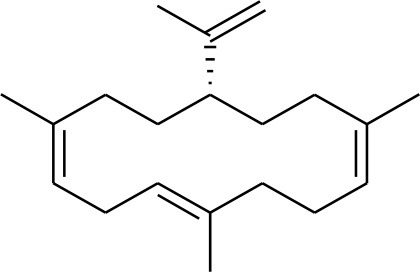	**Shmidt et al.**, **1970****; Patil et al.**, **1973**
**5**	Isocembrene	*Boswellia carterii*	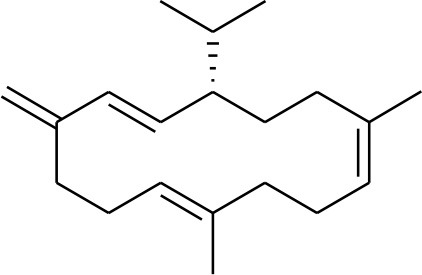	**Wahab et al.**, **1987**
**6**	Cembrene C	*Boswellia carterii*	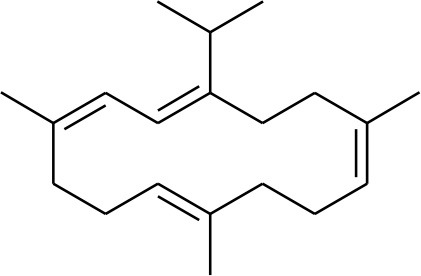	**Barakat and Rullkötter**, **1993**
**7**	Incensole oxide	*Boswellia carterii*	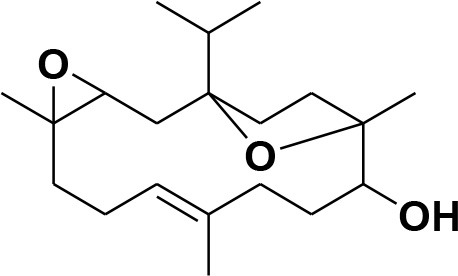	**Nicoletti and Forcellese**, **1968**
**8**	Incensole oxide acetate	*Boswellia carterii*	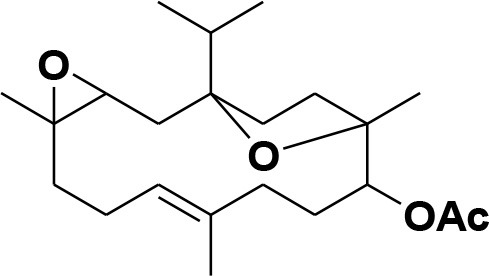	**Hamm et al.**, **2005**
**9**	Isoincensole oxide	*Boswellia carterii*	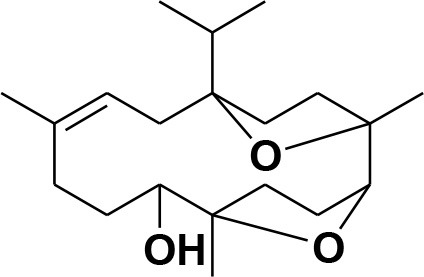	**Yamago et al.**, **1998**
**10**	Cembrene	*Boswellia carterii*	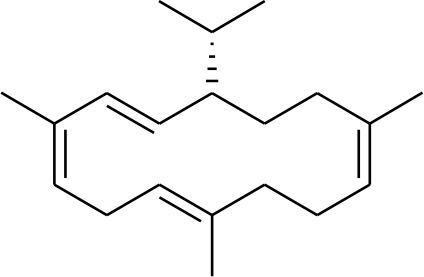	**Basar et al.**, **2001**
**11**	Isocembrene	*Boswellia carterii*	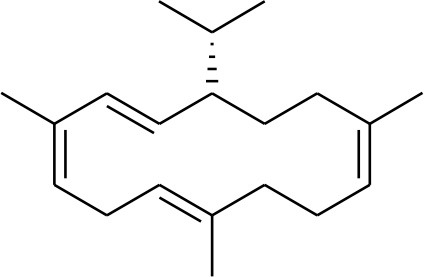	**Basar et al.**, **2001**
**12**	Thunbergol	*Boswellia carterii*	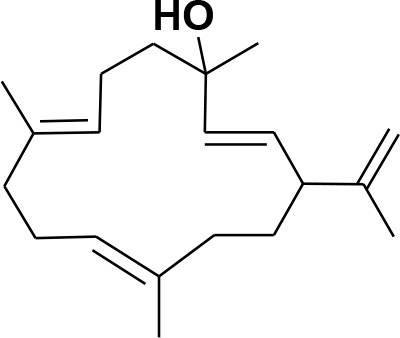	**Mikhaeil et al.**, **2003**
**13**	Duva-4,8,13-triene-1,3-α-diol	*Boswellia carterii*	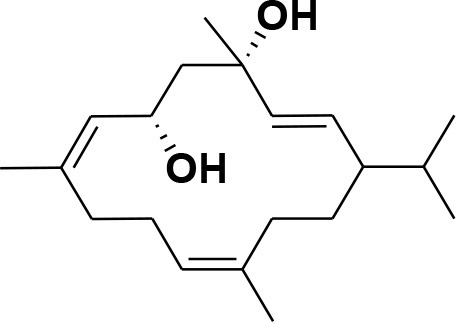	**Mikhaeil et al.**, **2003**
**14**	Duva-3,9,13-triene-1-α-hydroxy-5,8-oxide-1-acetate	*Boswellia carterii*	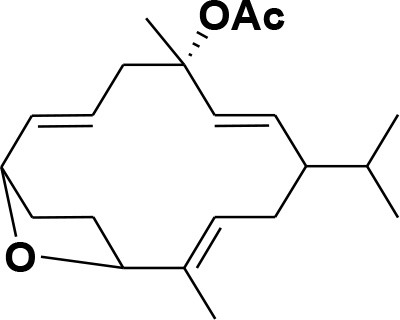	**Mikhaeil et al.**, **2003**
**15**	Duva-3,9,13-triene-1,5-α-diol- 1-acetate	*Boswellia carterii*	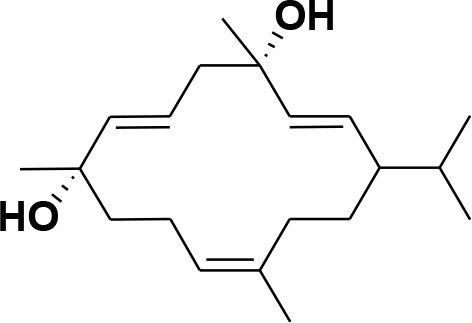	**Mikhaeil et al.**, **2003**
**16**	Duva-3,9,13-triene-1,5-α-diol	*Boswellia carterii*	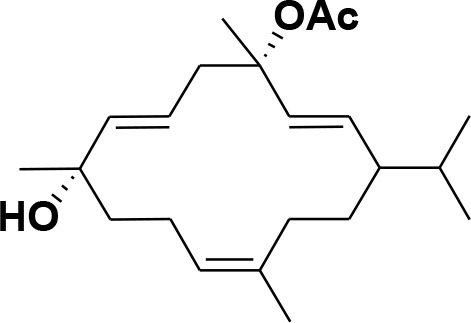	**Mikhaeil et al.**, **2003**
**17**	Boscartin A	*Boswellia carterii*	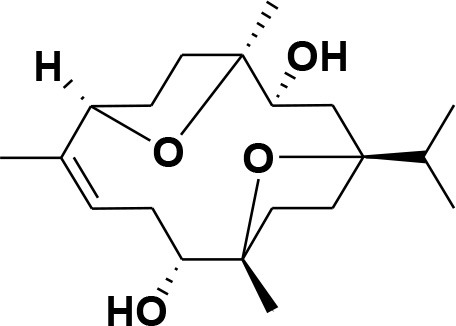	**Ren et al.**, **2015**
**18**	Boscartin B	*Boswellia carterii*	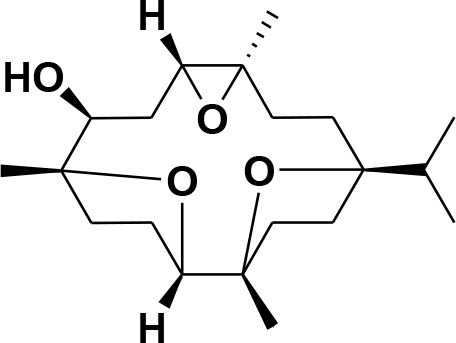	**Ren et al.**, **2015**
**19**	Boscartin C	*Boswellia carterii*	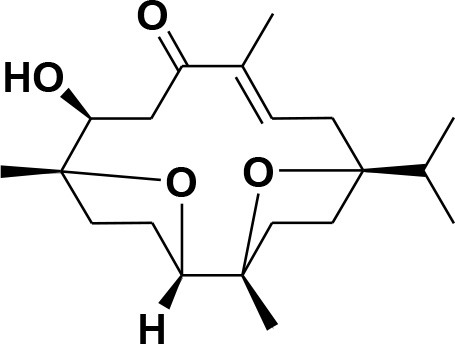	**Ren et al.**, **2015**
**20**	Boscartin D	*Boswellia carterii*	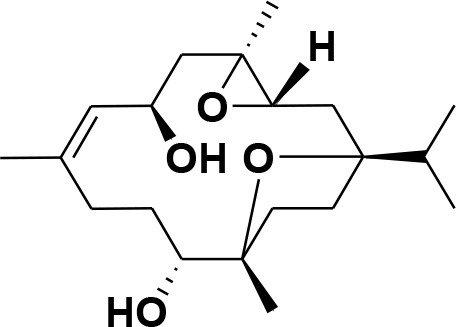	**Ren et al.**, **2015**
**21**	Boscartin E	*Boswellia carterii*	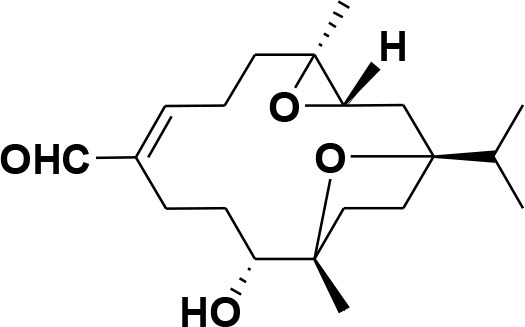	**Ren et al.**, **2015**
**22**	Boscartin F	*Boswellia 2carterii*	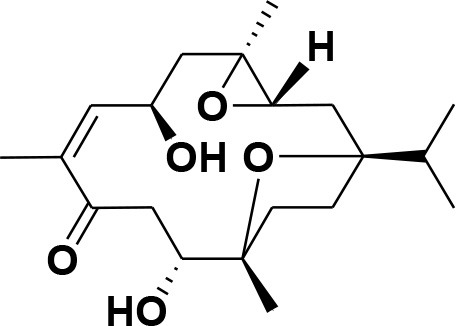	**Ren et al.**, **2015**
**23**	Boscartin G	*Boswellia carterii*	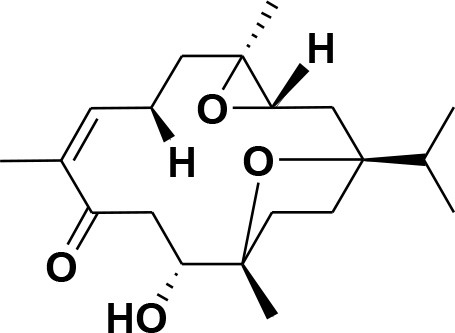	**Ren et al.**, **2015**
**24**	Boscartin H	*Boswellia carterii*	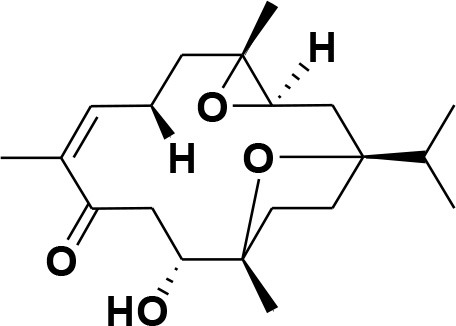	**Ren et al.**, **2015**
**25**	Iso-serratol	*Boswellia carterii*	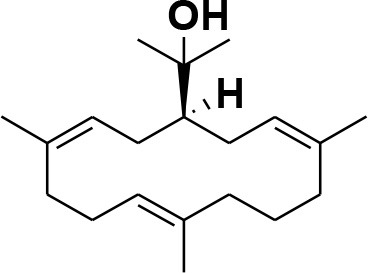	**Basar**, **2005**
**26**	Incensfuran	*Boswellia papyrifera*	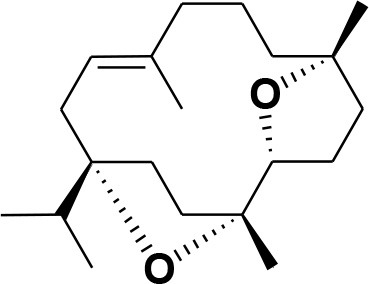	**Rehman et al.**, **2017**
**27**	Iso-incensolol	*Boswellia carterii*	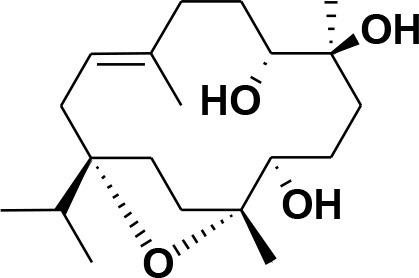	**Li et al.**, **2010**
**28**	β-Boswellic acid	*Boswellia sacra*	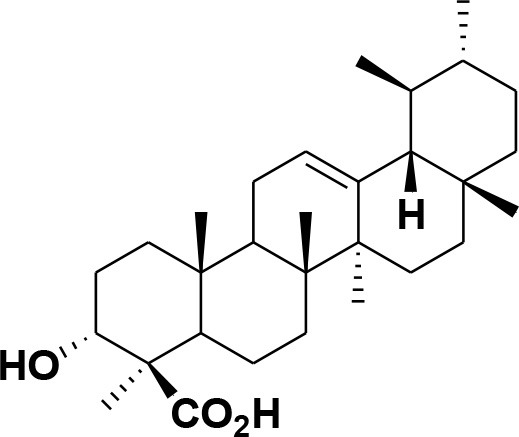	Pardhy, 1978; Al-Harrasi et al., 2018a
**29**	11-keto-β-Boswellic acid	*Boswellia sacra*	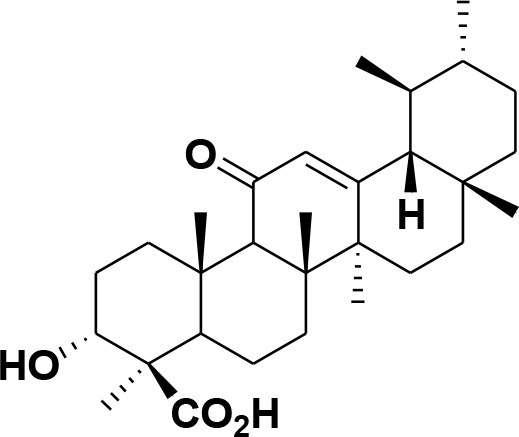	Pardhy, 1978; Al-Harrasi et al., 2018a
**30**	3-α-acetyl-β-Boswellic acid	*Boswellia sacra*	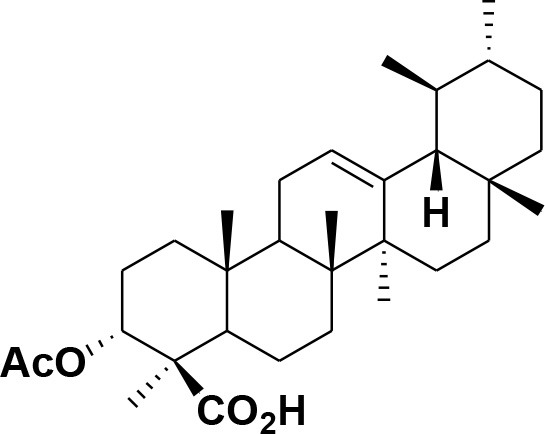	Pardhy, 1978; Al-Harrasi et al., 2018a
**31**	3-α-acetyl-11-keto-β-Boswellic acid	*Boswellia sacra*	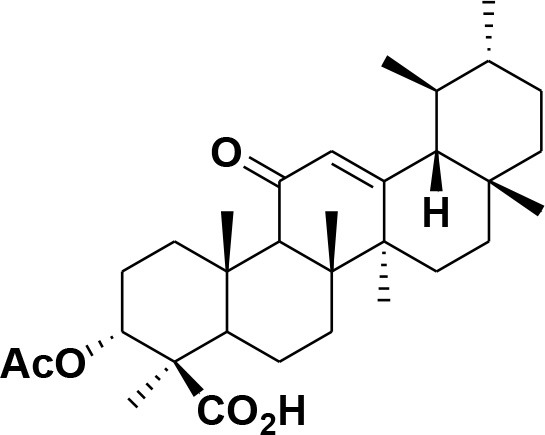	Pardhy, 1978; Al-Harrasi et al., 2018a
**32**	α-Boswellic acid	*Boswellia carterii*	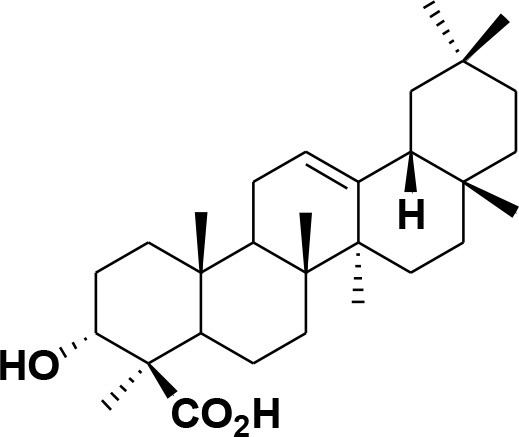	Akihisa et al., 2006
**33**	3- α -acetyl-α-Boswellic acid	*Boswellia carterii*	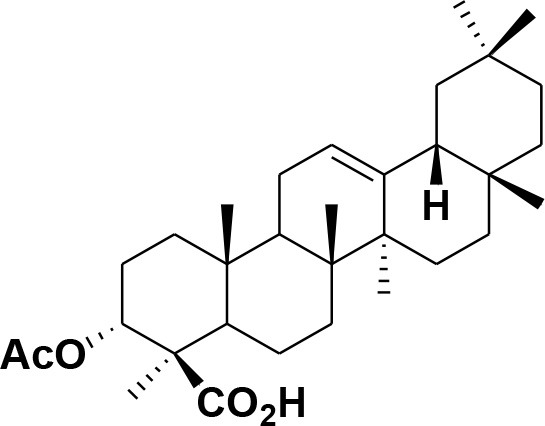	Akihisa et al., 2006
**34**	3,11-dihydroxy-12-ene-24-oic acids	*Boswellia sacra*	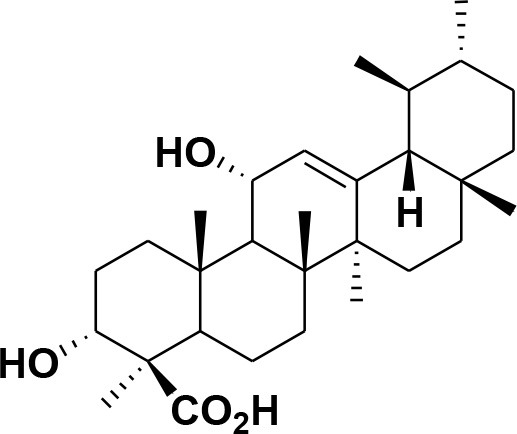	Al-Harrasi et al., 2018b
**35**	2a,3a-dihydroxy-urs-12-ene-24-oic acid	*Boswellia serrata*	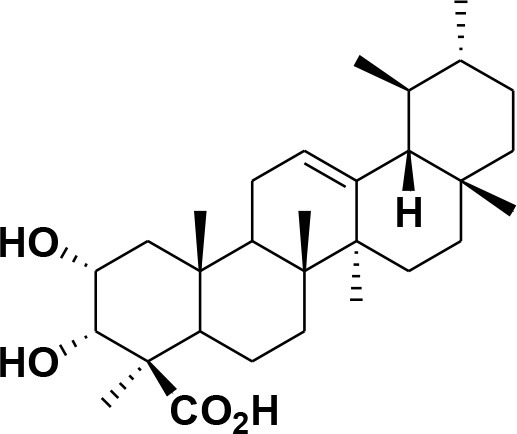	Mahajan et al., 1995
**36**	11-α-methoxy-β-boswellic acid	*Boswellia carterii*	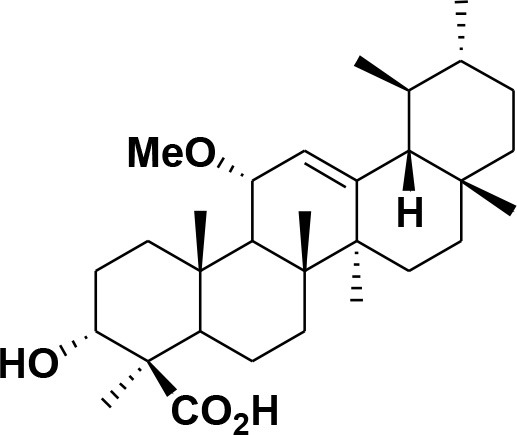	Ota and Houghton, 2008
**37**	11-α-ethoxy-β-boswellic acid	*Boswellia carterii*	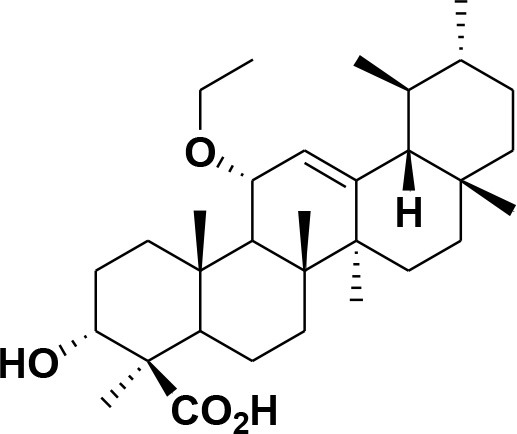	Al-Harrasi et al., 2013b
**38**	9,11-dehydro-β-boswellic acid	*Boswellia sacra*	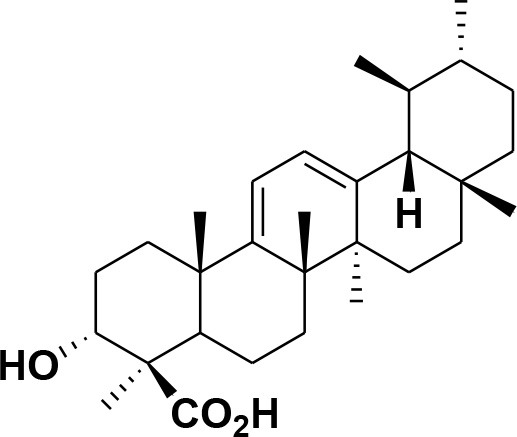	Ali et al., 2014
**39**	3-O-acetyl-9,11-dehydro-β- boswellic acid	*Boswellia sacra*	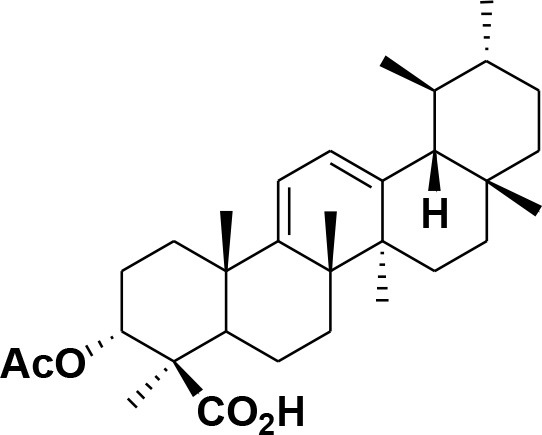	Al-Harrasi et al., 2013a
**40**	9,11-dehydro-α- boswellic acid	*Boswellia serrata*	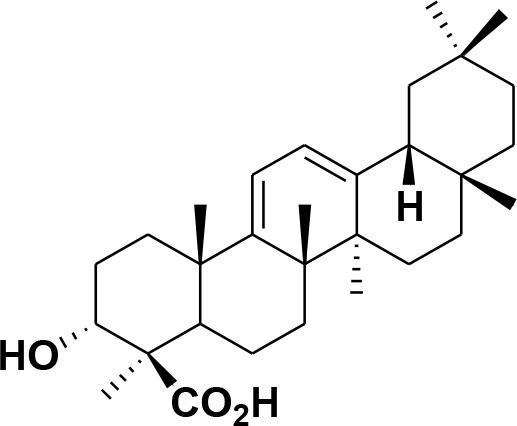	Büchele et al., 2003
**41**	3-O-acetyl-9,11-dehydro-α- boswellic acid	*Boswellia serrata*	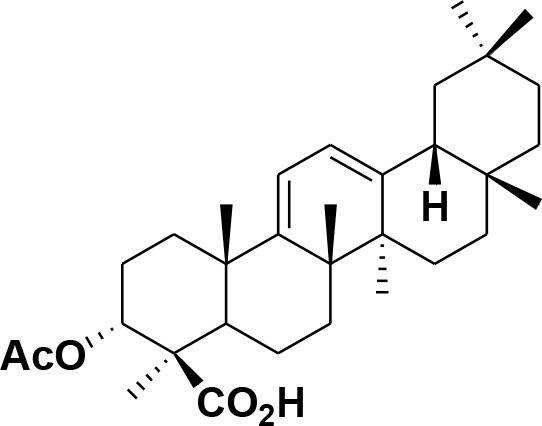	Büchele et al., 2003
**42**	3a-acetoxyurs-5:12-dien-24-oic acid	*Boswellia sacra*	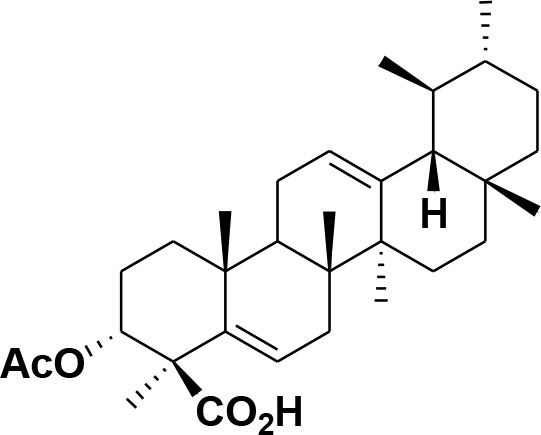	Ali et al., 2014
**43**	3-acetyl-11-hydroxy-12-ene-24-oic acids	Common Boswellia	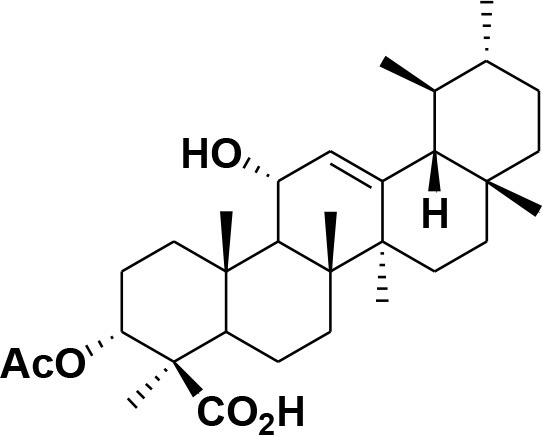	Corsano and Iavarone, 1964
**44**	3-epi-β-Boswellic acid	Synthetic	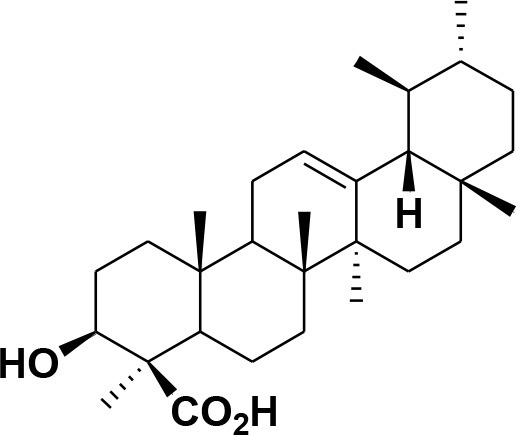	Kumar et al., 2012
**45**	3-epi-11-keto-β-Boswellic acid	Synthetic	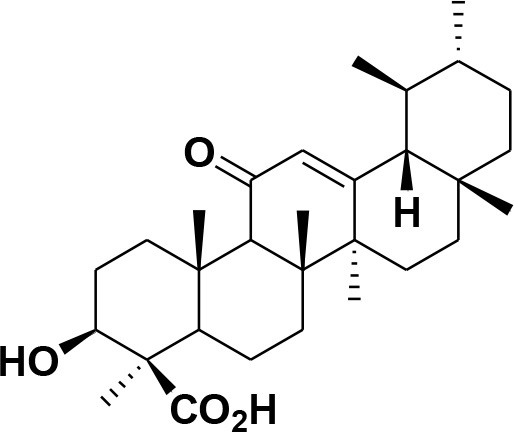	Kumar et al., 2012
**46**	3-epi-α-Boswellic acid	Synthetic	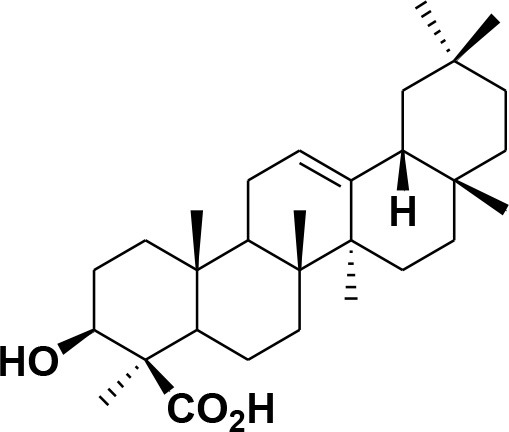	Al-Harrasi et al., 2018b

### Reverse Molecular Docking by MOE

A reverse docking approach was applied to search the appropriate druggable binders of macrocyclic diterpenoids and triterpenes by using MOE docking suit (Lee et al., 2016; Xu et al., 2018). After the preparation of protein and ligand files, docking was performed by using the Triangle Matcher docking algorithm and London dG scoring function. Induced Fit protocol (implemented in MOE) was applied during docking. The ligand binding residues were selected to define the active/ligand binding site (Table 1). By default, 30 docked conformations of each ligand were retained after docking. At the end of docking, the interactions of each ligand with the binding residues were visualized by Protein-ligand Interaction Fingerprints (PLIF) setup of MOE and Chimera. Later, conformational sampling was performed, and the best docked pose of each ligand was selected based on the docking score, rank, and binding interactions with the target. PLIF calculates hydrogen bonding, protein-ligand solvent bridging, ionic attraction, surface contact, metal ligation, arene attraction, and protein-ligand solvent interactions between protein and ligand. The 2D interactions of ligands with their targets were visualized by the MOE protein-ligand interaction tool. Subsequently, Chimera was used to depict the 3D view of protein-ligand interactions.

### Molegro Virtual Docker

For each docking, protein and ligand structures were imported into the MVD (version 2019.7.0.0) workspace in “pdb” and “mol2” format, respectively (Thomsen and Christensen, 2006). The ligands were prepared by MOE by applying charges and explicit hydrogen, and the valences, bond orders, and protons were thus correct in the ligands. The ligand binding site in the protein was defined by cavity detection algorithm of MVD. Thirty docking runs were performed for each ligand with a maximum iteration of 1,500 and maximum population size of 50, which resulted in 30 docked poses of each ligand after docking. Later, the compounds were ranked on the basis of MolDock and re-rank scores.

### AutoDock Vina

AutoDock Vina (version 1.1.2) was used in this study (Trott and Olson, 2010). AutoDock Vina requires the receptor and the ligand files in PDBQT [Protein Data Bank, Partial Charge (Q) and Atom Type (T)] format. We used the same PDB files of the receptors and the mol2 files of the ligands that were used in MOE, and we converted the structures to pdbqt format using PyRx Virtual Screening tool [https://pyrx.sourceforge.io/]. The docking search space was defined around 3 Å of selected residues or ligand (where available) with a grid box size of 25 × 25 × 25 Å. The number of binding modes and exhaustiveness of search was set to 30 and eight, respectively. Later, each of the conformation was visualized on UCSF Chimera, and the best docked orientation was selected based on docking score and binding interactions with the protein.

### Analysis Measures and Conformational Sampling

To select the most appropriate inhibitor against selected target, a consensus approach was used. The docked library was sorted on the basis of docking scores, and the suitable inhibitor for a particular target was selected when it is ranked among the top 10 ligands of the screened dataset by two out of three programs. The most optimal binding mode of each compound was chosen by conformational sampling. All the docked poses of each compound (generated by each program) were compared, and the binding mode that was analogous in all the docking methods was declared as the probable binding mode.

## Results and Discussion

### Re-docking

The robustness of the docking methods was scrutinized by re-docking experiments. For this purpose, 10 (IL-17, IL-36γ, PPAR-γ, MAPK2, JAK1/2/3, TNF-α, iNOS, and eNOS) out of 18 selected targets were chosen due to the presence of co-crystallized ligands (inhibitors) in their PDB structures. All the programs successfully re-produced the X-ray confirmed orientation of each ligand with root mean square deviation (RMSD) of ≤2.5 Å. Additionally, the screening reliability was justified by the ranking position of known inhibitors of all the target, and it was observed that all the positive controls were ranked at the top of the screened library. The results indicate that the used docking methods are reliable in predicting the binding orientation and in the selection of known inhibitors. The re-docking results are summarized in supporting information Table S1.

### Molecular Targeting of Cembrane Diterpenes and Boswellia Triterpenes

In the current study, a reverse docking approach was employed to predict the binding potential of selected cembrenoid diterpenoids and *Boswellia* triterpenoids with the 18 drug targets. For this purpose, three different docking algorithms and scoring functions were used. Those proteins, which were collectively ranked with higher score by all the docking methods, were considered as “best” targets. Based on the docking score, JAK1/2/3, eNOS, and iNOS were proposed as the best targets for all the compound by MOE and ADT vina, while MVD only picked 17/46 and 12/46 compounds as excellent inhibitors for JAK1 and eNOS, respectively. Similarly, ADT Vina ranked JAK1 and eNOS among the top ranked targets for all the compounds, while JAK2, JAK3, and iNOS were retrieved as good binders for 42/46, 40/46, and 39/46 compounds, respectively. MVD demonstrated that 40/46, and 37/46 compounds can efficiently target JAK2 and 3, respectively. While >39, 26, and 50% of the screened library may target JAK1, eNOS, and iNOS, respectively. Thus, JAK1/2/3, eNOS and iNOS were considered as the most appropriate druggable candidates for the selected di- and tri- terpenoids.

Among the selected cytokines, IL-17 was retrieved as an interesting target since MVD showed that all the compounds may neatly fit at the binding pocket of IL-17, while MOE and ADT Vina suggested that 42/46 and 40/46 compounds can effectively target IL-17, respectively. According to MOE and ADT Vina, 45/46 compounds possess high to moderate binding affinities for TNF-α, while MVD showed that >63% of the screened database holds good inhibitory potential against TNF-α. Therefore, IL-17 and TNF-α were also considered as good targets for these compounds.

**Figure 1:**
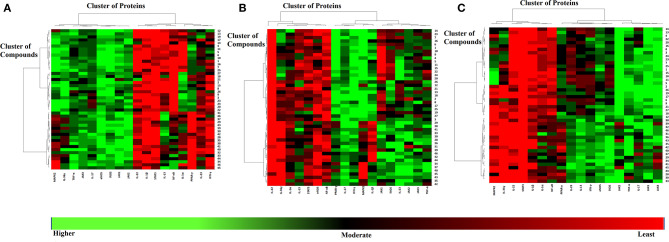
The graphical presentation of binding potential of all the compounds with the selected targets as a Heat Map. The results of MOE **(A)**, MVD **(B)**, and ADT Vina **(C)** are shown. The red to green through black scale was used to demonstrate the binding affinities of compounds toward each target where red color indicates the least potential, black shows moderate binding and green indicates highest binding potential. The clustering of compounds is shown on Y-axis, while the clustering of targets is shown in X-axis.

Based on the *in-silico* outcomes, JAK1/2/3, eNOS, iNOS, IL-17, and TNF-α are the most appropriate targets for the purified chemical constituents of frankincense. Moreover, the consensus results of all the docking methods depicted that MAPK2, PPAR-γ, IL-13, IL-23, IL-36γ, and IFNγ can serve as possible binding proteins for several di- and tri- terpenes. Meanwhile, IL-1α, IL-1β, NF-κB, IL-22, and STAT3 were identified as the least potential targets for these cembrenoid derivatives. The best inhibitors for each target are given in supporting information Table S2 and the docking scores are tabulated in Tables S3–S5. The predicted binding potential of each compound with the selected target is presented as a heatmap in Figure 1.

### Top Predicted Targets

#### JAK1/2/3

**Figure 2:**
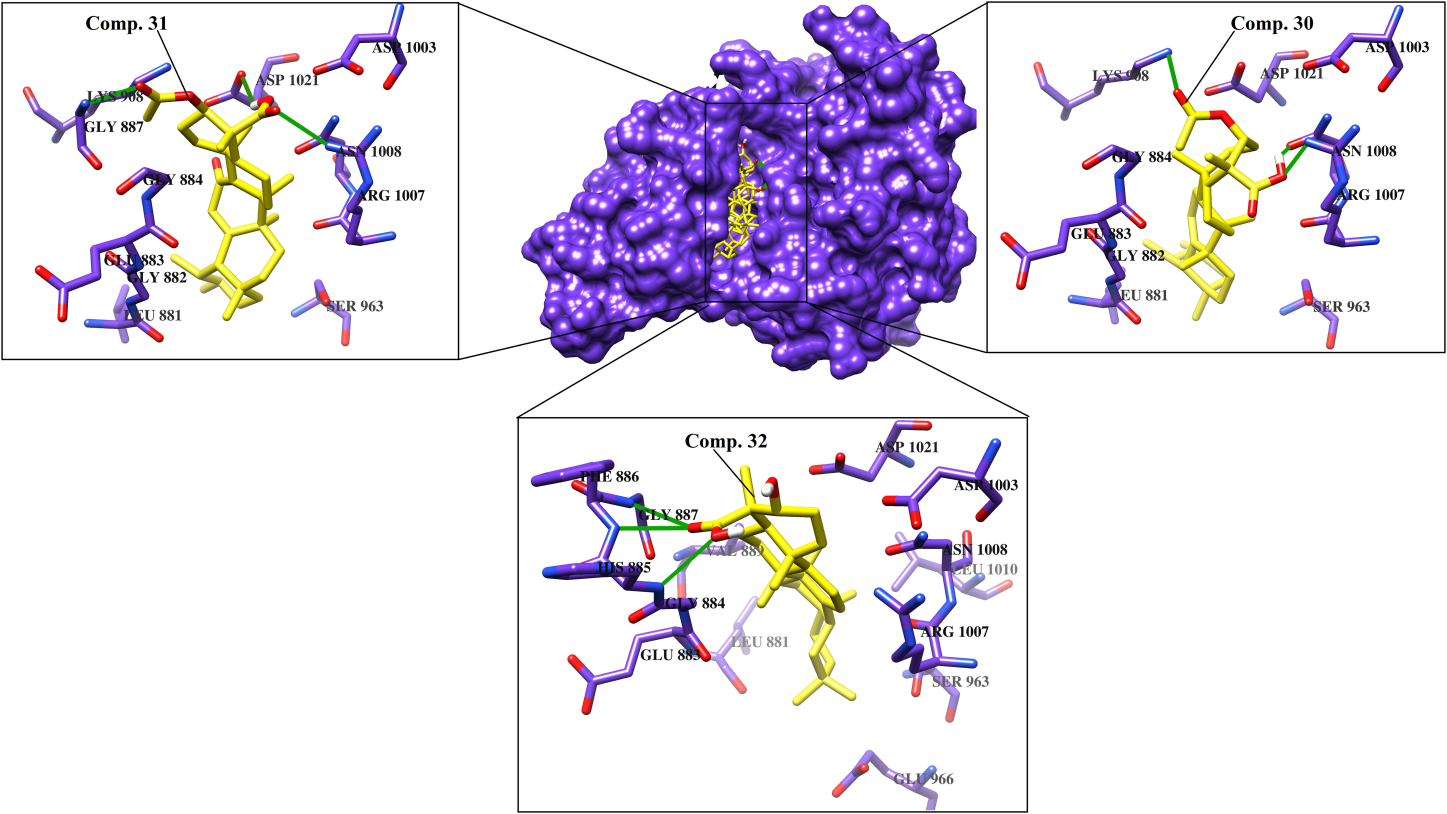
The docked view of the most active compounds **30**–**32** in the binding site of JAK1 (PDB code: 6N7B). The protein and active site residues are shown in purple surface and stick models, respectively. The entrance of the active site is highlighted in the box. The inhibitors are presented in a yellow stick model, and the Hydrogen bonds are displayed in green lines.

Various cytokines are regulated by the JAK-STAT pathway, particularly TH17 signaling, which is important in the pathogenesis of psoriasis. JAK inhibitors are efficacious against psoriasis, alopecia areata, and atopic dermatitis (Ciechanowicz et al., 2019). When docked at the active site of JAK1, all the di- and tri- terpenoids were predicted as the potent inhibitors by MOE and ADT vina; however, MVD ranked 17/46 compounds (**12, 28-34, 36-38, 40-41**, and **43-46**) as the best binders of JAK1. Among them, four molecules (**6, 30-32**) were mutually ranked by all the docking methods among the top 10 ligands; however, interaction analysis suggest that only **30**-**32** can serve as potent inhibitor of JAK1. The binding modes of **30**-**32** suggests that these compounds neatly fit at the active site of JAK1 where Lys908, Arg1007, Asn1008, Phe886, His885, and Gly887 particularly stabilizes these compounds through H-bonding. The carboxylic group of 3-α-acetyl-β-Boswellic acid (**30**) mediates bidentate interactions with the side chains of Asn1008 and Arg1007, while the acetyl moiety interacts with the side chain of Lys908. Similarly, carboxylic group of 3-α-acetyl-11-keto-β-Boswellic acid (**31**) interacts with the side chains of Asn1008 and Asp1021, whereas the acetyl group mediates H-bonding with the amino group of Gly887. However, the carboxylic group of α-Boswellic acid (**32**) mediates H-bonding with the amino groups of His885, Phe886, and Gly887. These pentacyclic triterpenic boswellic acids possesses promising anti-inflammatory properties (Al-Harrasi et al., 2018a). The docked conformations of **30**-**32** are presented in Figure 2.

JAK2 and JAK3 were also identified as superior targets for each molecule by MOE, while MVD and ADT vina demonstrated that 40/46 compounds can effectively target JAK2. The consensus examination depicted that five cembrane diterpenes (**3, 14, 16, 20, 23**,) and three boswellic acid derivatives (**28, 37**, and **43**) are the most prominent inhibitor of JAK2. The binding mode of incensole acetate (**3**) showed that the acetate moiety of the compound mediates H-bonding with the side chain of Lys882, which is homologous to Lys908 and Lys855 in JAK1 and JAK3, respectively. Lys882 also mediate H-bonding with the acetate groups of **14** and**16**. The docked view of **20** reflects that this compound has a chance to bind with several residues (Leu932, Tyr931, and Arg980) due to the presence of several polar -OH groups in its structure. The -OH and the carbonyl groups of **23** interact with the side chains of Lys882 and Asp994, respectively. This indicates that Lys882 is a crucial residues in the stabilization of diterpenoids at the active site of JAK2. The carboxylic groups of triterpenoid **28** and **43** were found to be H-bonded with the side chain of Arg980, while the carboxylic moiety of **37** interacts with the side chain of Asn981 *via* H-bonding. Arg980 and Asn981 of JAK2 are homologous to Arg1007 and Asn1008 of JAK1, respectively. This suggest that these Arg and Asn residues play important roles in the binding of boswellic acids in the active site of JAK1/2.

**Figure 3:**
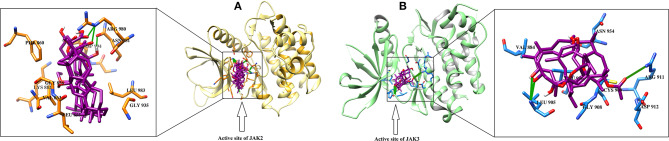
The docked view of the predicted most active compounds in the active site of **(A)** JAK2 and **(B)** JAK3. The active site residues of JAK2 and JAK3 are presented in the orange and blue stick model, respectively. The ligands (**28, 37**, and **43** in the active site of JAK2 and **16**, **20**, and **24** in the active site of JAK3) are shown in magenta stick model, and the H-bonds are displayed in green lines.

For JAK3, only 9/46 and 6/46 compounds showed the least inhibitory potential in MVD and ADT vina docking, respectively, while rest of the compounds exhibited higher binding potential. Meanwhile, compounds **16, 20, 24**, and **36** were communally ranked at the top of the screened dataset by all three scoring functions. It was observed that compounds **16** and **20** can inhibit both the enzymes. 3-α-acetyl-11-keto-β-boswellic acid (**31**) is known to inhibit the activation of STAT3 by inhibiting the phosphorylation of JAK2 and Src. For the dephosphorylation of STAT3, compound **31** induces Src homology region 2 domain-containing phosphatase 1 (SHP-1) (Kunnumakkara et al., 2009). Our docking results are consistent with these experimental findings. Compound **31** was identified as a good inhibitor of JAK1/2/3 by all the scoring functions. The binding modes of selected hits (**16, 20, 24**, and **36**) showed that the methyl acetate of **16** binds with Arg911 and Cys909, while the -OH group of **20** and the carbonyl group of **24** interacts with the amino group of Leu905. The binding mode of 11-α-methoxy-β-boswellic acid (**31**) depicted that this compound did not produce significant binding interactions within the active site of JAK3. The binding modes of best predicted inhibitors in the active site of JAK2/3 are shown in Figure 3.

#### eNOS and iNOS

**Figure 4:**
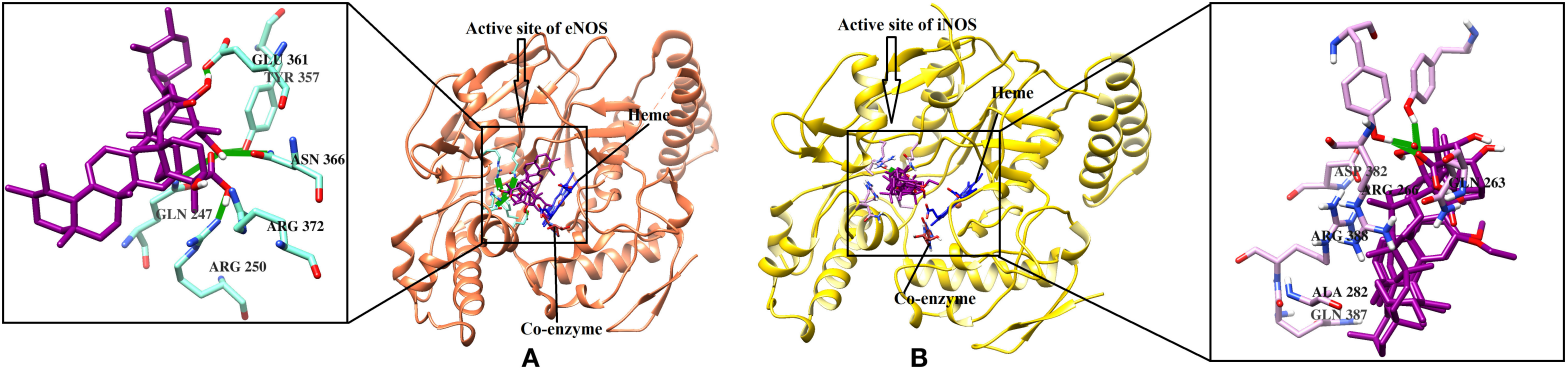
**(A)** The 3D-structure of eNOS (PDB code 4D1P) is shown in a complex with heme, a coenzyme and compounds **28–29 (B)**. The molecular structure of iNOS (PDB code 3E7G) is shown in complex with heme, and compounds **36-37, 40**. Heme and co-enzyme moieties are shown in blue and brown stick model, respectively. The H-bonds are shown in green lines. The ligands are depicted in magenta stick model. The binding residues of each enzyme are highlighted in box.

eNOS and iNOS are involved in immune response, and they are important enzymes in the pathogenesis of psoriasis (Mittal et al., 2009; Rácz and Prens, 2009). The post docking analysis showed that all the compounds bind at the entrance of the active site of eNOS and iNOS. All the derivatives were ranked as good inhibitors of eNOS and iNOS by MOE. Similarly, ADT vina also showed that all the cembrenoids possess greater inhibitory affinities for eNOS; however, except for **1, 2, 5, 7, 10, 11**, and **15**, the rest of the compounds exhibits excellent binding tendency for iNOS. Despite this, the MolDock score filtered only few compounds as good binders of eNOS (Table S2) and 50% of the docked library (**2, 3, 8, 12, 13, 18, 21, 26, 29, 31–34, 36-38**, and **40–46**) for iNOS. Among all, compounds **15–16, 28–29**, and **37–38** as well as compounds **8, 21, 36–37**, and **40** were of utmost importance for eNOS and iNOS, respectively. It is shown that compound **37** targets both eNOS and iNOS effectively, suggesting it could be the most important inhibitor of eNOS and iNOS. The binding modes of the most active compound **28–29** and **36–37** and **40** in the active site of eNOS and iNOS, respectively, are shown in Figure 4.

#### IL-17

IL-17 and TNF-α emerged as good targets for most of the compounds by all the docking methods. An IL-17 dimer binds with IL-17RA and forms a protein-protein interface, which is also blocked by recently identified cyclo-/linear- peptide inhibitors (Blauvelt and Chiricozzi, 2018; Brembilla et al., 2018). Based on the CA criterion, compounds **16, 18, 28, 29**, and **32** were retrieved as the most promising inhibitors for IL-17. The mode of interactions reveals that **18, 28, 29**, and **32** are well accommodated at the antagonist binding site of IL-2 where Gln94, Glu95, and Leu97 play important roles in the stabilization of these compounds (supporting information Figure S1).

#### TNF-α

TNF-α plays a crucial role in the exacerbation of inflammation in psoriasis. The management of psoriasis is done by inhibiting the production of TNF-α by several inhibitors like adalimumab, certolizumab pegol, etanercept, golimumab, and infliximab. MOE and ADT vina ranked TNF-α as a moderate binder for all the compounds except compound **26** and **30**, respectively. While MVD predicted that 63% of compounds can exhibit good-to-moderate inhibition of TNF-α. According to CA, compounds **20, 36, 38, 40, 43**, and **45** were regarded as the most effective binders of TNF-α. mediated favorable interactions with the Ser60, Leu120, Gly121, and Tyr151 and displayed excellent to moderate inhibition of TNF-α *in silico*. The interactions of ligands with TNF-α are shown in Figure S2.

### Good Targets for Several Cembrane Diterpenes and Boswellia Triterpenes

#### MAPK2, PPAR-γ, IL-13, IL-23, IL-36γ, and IFNγ

Several di- and tri terpene derivatives exhibited excellent binding affinities for MAPK2, PPAR-γ, IL-13, IL-23, IL-36γ, and IFNγ in our molecular docking experiments (Table S2).

#### MAPK2

In psoriasis, the activation of MAPK2 causes epidermal hyperproliferation (Johansen et al., 2006). MOE, MVD, and ADT vina ranked 29/46, 28/46, and 11/46 compounds as good inhibitors of MAPK2, respectively. Among the docked compounds, **14-17, 20, 22-24**, exhibited excellent binding affinities within the active site of MAPK2.

#### PPAR-γ

PPAR-γ regulates lipid and glucose metabolism. Recently, the pathophysiological role of PPAR-γ was reported in the inflammatory immune response, and it was identified as a major drug target for the treatment of psoriasis, benign epidermal tumors, and atopic dermatitis (Sertznig and Reichrath, 2011). The docked compounds at the active site of PPAR-γ demonstrated that **9–11, 15–16, 18, 20**, and **22** hold greater binding affinities with PPAR-γ. Among the selected hits, compounds **15** and **20** are excellent inhibitors of PPAR-γ. Liu et al. (2013) described that compound **31** down-regulates PPAR-γ2 expression and loss of phenotypic markers of mature human adipocytes and mobilizes lipolysis. MVD and ADT vina placed compound **31** in the list of good inhibitors (Liu et al., 2013).

#### Interleukin-36γ

IL-36γ binds with its receptor (IL-36RA) to produce immune response and found to be upregulated in psoriatic and dermatitis skin (D'erme et al., 2015; Braegelmann et al., 2016). The diterpenoids and boswellic acid derivatives were docked at the recently identified antagonist binding site of IL-36γ, which is also a binding site for the IL-36γ receptor. According to the docking scores, MOE, MVD, and ADT vina detected IL-36γ as a good target for 35, eleven, and eight compounds, respectively. The compounds **7, 13-16, 20**, and **22–24** displayed highest potency against IL-36γ. Interestingly, the diterpenoids displayed more binding potential than triterpenoids for MAPK2, PPAR-γ and IL-36γ.

#### Interleukin-13

IL-13 binds with its receptors IL4R/IL13Rα1 and IL-13Rα2 to trigger allergic response. IL-13 and its receptors (IL4R/IL13Rα1, IL-13Rα2) are upregulated in psoriatic skin and lesions (Baliwag et al., 2015). IL-13 in complex with its receptor (IL-13Rα2) was chosen for docking, and the compounds were directed at theIL-13Rα2 binding site, where 11/23 (**28–31, 35, 37–39, 42–43**, and **46**) 17/23 (**4, 9, 12, 19, 26, 29, 33–34, 37–43**, and **45–46**), and 22/46 (**12, 19, 24, and 28–46**) compounds demonstrated good binding potential by MOE, MVD, and ADT vina, respectively. Among these molecules, 11-α-ethoxy-β-boswellic acid (**37**) was retrieved as the most prominent hit, however compounds **28, 30, 31, 39, 45**, and **46** were ranked among the top 10 ligands by MOE and ADT vina.

#### Interleukin-23

IL-23 is a heterodimeric cytokine, composed of IL-23p19 (p19 subunit) and IL-12p40 (p40 subunit), which binds with its receptor (IL-23R), and the IL-12 receptor subunit β1 (IL-12β1) for the signaling. The compounds were directed at the IL-23/IL23R binding site. The N-terminal domain of p19 subunit of IL-23 binds with the N-terminal domain of IL23R where Arg57, Glu58, Trp156, and Lys164 of IL-23 interacts with Glu111, Thr112, Asp118, Gly24, Asn27, Asn29, and Leu113 of IL-23R. After docking, MVD, MOE, and ADT vina chose 36, six, and 27 compounds as good inhibitors of IL-23, respectively. However, five compounds, i.e., Boscartin F (**22**), 3-α-acetyl-11-keto-β-Boswellic acid (**31**), 3-O-acetyl-9,11-dehydro-β- boswellic acid (**39**), 3-epi-β-Boswellic acid (**44**), and 3-epi-11-keto-β-Boswellic acid (**45**) were identified as novel and potent inhibitors of IL-23.

#### Interferon γ

IFNγ is involved in the innate and adaptive immunity against viral and some bacterial infections. Elevated level of IFNγ has been observed in the serum of psoriatic patients (Abdallah et al., 2009). MVD showed that all the compounds can target IFNγ-Rα binding site, while MOE and ADT vina retrieved six and 26 molecules as good inhibitors of IFNγ, respectively. The false positives were removed by consensus results, which showed that five compounds (**17, 20, 22, 39**, and **46)** are the most prominent inhibitors of IFNγ.

## Discussion

The importance of the immune system in the pathogenesis of psoriasis is revealed by efficacy of several immunosuppressive agents, including cyclosporine, denileukin diftitox, and alefacept. TNF-α is usually elevated in psoriatic lesions; however, overexpression of several other cytokines, such as IL-13, IL-17, IL-22, IL-36γ, and IFN-γ, has also been observed in the skin cells of psoriatic patients. These cytokines mediate the potentiation of keratinocytes on psoriatic inflammation, and the use of antibodies against IL-23, TNF-α, and IL-17 in the treatment of psoriasis has shown a key role of these cytokines in the pathogenesis of psoriasis (Lowes et al., 2014; Harden et al., 2015). Moreover, studies have confirmed that the NF-κB pathway is activated in psoriatic lesions and is downregulated after successful treatment (Wang et al., 2017). Furthermore, several other proteins, including IL-1α/β, eNOS, iNOS, PPAR-γ, MAPK2, JAK1/2/3, and STAT3, were identified as possible novel therapeutic targets, which are overexpressed in psoriasis patients (Mittal et al., 2009; Rácz and Prens, 2009).

It is reported that serratol (**1**) and incensole acetate (**3**) inhibit the expression of TNF-α, NF-κB, and IL-1β (Moussaieff et al., 2008). Several derivatives of cembrenoid diterpenoids and boswellic acid are well known for their inhibitory activities against the production of TNF-α (Al-Harrasi et al., 2018a). Our docking results are consistent with those experimental findings. The transcription factor NF-κB orchestrates inflammation and regulates the activity of several proinflammatory genes. NF-κB serves as a crucial mediator in the pathogenesis of psoriasis (Goldminz et al., 2013; Moorchung et al., 2014). Several studies showed that incensole (**2**) and incensole acetate (**3**) suppress the activation of NF-κB by inhibiting TAK/TAB-mediated IκB kinase (IKK) activation loop phosphorylation (Berrrrpohl et al., 2007; Moussaieff et al., 2007). It is also reported that **3** did not suppress IκBα phosphorylation in co-stimulated T cells, indicating that the kinase inhibition is neither direct nor does it affect all NF-κB activation pathways. Moreover, acetyl-11-keto-β-boswellic acid (**31**) and acetyl-α-boswellic acid (**33**) inhibits NF-κB signaling by specifically inhibiting IκBα kinase (IKK), which is pivotal for the degradation of the NF-κB inhibitor IκB, as well as the phosphorylation of p65, two steps essential for NF-κB activation and the subsequent cytokine expression. Syrovets et al. (2005) proved that acetyl-boswellic acids inhibits LPS-mediated TNF-α induction in monocytes by direct interaction with IκB kinase (Syrovets et al., 2005). Our docking results also indicate that these compounds have less of a potential to directly bind with NF-κB.

IL-17 is a pro-inflammatory cytokine produced by Th-17 cell in response to their stimulation by IL-23. IL-17 interacts with its receptor to mediate proinflammatory and allergic responses, and, as a result, production of several cytokines (IL-1β, TGF-β, TNF-α, IL-6, GM-CSF, and G-CSF), chemokines (GRO-α, IL-8, and MCP-1), and prostaglandins (PGE2) occurs via macrophages, keratinocytes, fibroblasts, epithelial, and endothelial cells. Activation of IL-17 signaling is observed in the pathogenesis of various autoimmune disorders, including rheumatoid arthritis, asthma, lupus, allograft rejection, anti-tumor immunity, psoriasis, and multiple sclerosis, and IL-17 inhibitors are being investigated as possible treatments for these disorders. The monoclonal antibodies (mAB) secukinumab and ixekizumab inhibit IL-17 while brodalumab blocks the IL-17 receptor (IL-17RA). By interfering with IL-17 signaling, these antibodies interrupt the inflammatory cycle of psoriasis. The mAB against IL-23 (ustekinumab) is also used to treat psoriasis by reducing IL-17, and, thus, the IL-23/IL-17 pathway plays a major role in psoriasis. Recently, Stürner et al. (2014) revealed that compound **29** (11-keto-β-Boswellic acid, KBA) reduces the differentiation of human CD4+ T cells to Th17 cells while slightly increasing Th2- and Treg-cell differentiation. Furthermore, KBA reduces the IL-1β-triggered IL-17A release of memory Th17 cells. KBA may affect IL-1β signaling by preventing IL-1R-associated kinase 1 phosphorylation and subsequently decreasing STAT3 phosphorylation at Ser727, which is required for Th17-cell differentiation. The effects of KBA on Th17 differentiation and IL-17A release make the compound a good candidate for potential treatment of Th17-driven diseases. Boswellic acids reduce Th17 differentiation via blockade of IL-1β-mediated IRAK1 signaling (Stürner et al., 2014). Our docking results also confirm that compound **29** (KBA) possesses excellent binding affinity for IL-17.

The monoclonal antibody (ustekinumab) inhibits IL-12/IL-23, which are both associated with psoriatic inflammation and psoriatic arthritis. Moreover, mAB against IL-23 (tildrakizumab, Risankizumab, and guselkumab) also reduce psoriatic symptoms and slow disease progression. IL-23 is involved in the proliferation of CD4+ Th17 cells to produce IL-17 and along with IL-17, associated with several autoimmune and inflammatory disorders such as psoriasis, psoriatic/rheumatoid arthritis, multiple sclerosis, Crohn's disease, uveitis, and inflammatory bowel disease (Braegelmann et al., 2016; Chan et al., 2018; Fotiadou et al., 2018). Boscartin F (**22**), 3-α-acetyl-11-keto-β-Boswellic acid (AKBA, **31**), 3-O-acetyl-9,11-dehydro-β- boswellic acid (**39**), 3-epi-β-Boswellic acid (**44**), and 3-epi-11-keto-β-Boswellic acid (**45**) were identified as excellent inhibitors of IL-23 in this study. Previously, the effect of **31** (3-α-acetyl-11-keto-β-Boswellic acid) was studied on activated dendritic cells in psoriasis-like mouse model that revealed that this compound improved the psoriasis-like skin lesions, reduced the thickness of epidermis, ameliorated the infiltration of CD3+ and CD11c+ cells in skin lesions, decreased the activation of local dendritic cells, inhibited the expression and secretion of IL-12 and IL-23, inhibited the maturation and differentiation of DCs to promote T-cell differentiation, and inhibited the activation of TLR7/8 and IRF signaling pathways. Thus, AKBA might have an anti-inflammatory effect on psoriasis by inhibiting the maturation and activation of DCs via the TLR8 and IRF signaling pathways (Wang et al., 2018). Our docking results also suggests that AKBA tends to inhibit IL-23.

The cytokine IL-1α is produced by macrophages, neutrophils, and epithelial and endothelial cells, while IL-1β is produced by activated macrophages and mediates inflammatory response after binding with their receptor. Both the cytokines are excessively produced in psoriatic lesions (Tsai and Tsai, 2017). Both the cytokines were depicted as moderate to least binding proteins for this set of compounds. IL-22 is produced by T cells and is involved in the modulation of tissue responses during inflammation and found significantly higher in psoriatic patients (Fujita, 2013; Hofny et al., 2017; Voglis et al., 2018). IL-22 was also depicted as a least possible target for di- and tri-terpene derivatives.

PPAR-γ regulates lipid and glucose metabolism. Recently, the pathophysiological role of PPAR-γ was reported in the inflammatory immune response and identified as a major drug target for the treatment of psoriasis, benign epidermal tumors, and atopic dermatitis (Sertznig and Reichrath, 2011). Liu et al. (2013) described that AKBA (**31**) downregulates the expression of PPAR-γ2 and loss of phenotypic markers of mature human adipocytes and mobilizes lipolysis (Liu et al., 2013). In the current study, **31** was recognized as an excellent inhibitor of PPAR-γ by MVD.

In psoriatic patients, STAT3 is activated in lesional keratinocytes, which can lead to the development of regenerative epidermal phenotype observed in psoriasis (Sano et al., 2005). Incensole (**2**) is a potent inhibitor of STAT3 (Pollastro et al., 2016). It is reported that **29** (KBA) inhibits the phosphorylation of STAT3 (Stürner et al., 2014). In this study, both the compounds were identified as moderate inhibitors of STAT3 by MVD. Our *in-silico* experiment correlates well with the reported experimental findings. The docking studies are used in the discovery of novel inhibitors against several therapeutic targets (Halim et al., 2013). However, docking must be combined with more sophisticated computational techniques like molecular dynamic simulation to enhance the efficiency of virtual screening. This work needs to be further explored by molecular dynamic simulation to study the dynamic behavior of protein upon protein-ligand complex formation.

## Conclusion

The anti-psoriatic potential of several *Boswellia* diterpenoids and triterpenoids was explored by computational analysis. The compounds have displayed selective docking to human anti-inflammatory (JAK1/2/3, eNOS, iNOS, and TNF-α) and anti-psoriatic (IL-17) molecular targets. Moreover, several other proteins (MAPK2, PPAR-γ, IL-13, IL-23, IL-36γ, and IFNγ) were retrieved as leading biological targets for these compounds. Thus, these compounds have reflected excellent anti-psoriatic and anti-inflammatory potency *in silico*. The results indicate that *Boswellia* di- and tri-terpenoids can serve as promising chemical scaffolds for the development and improvement of inhibitors to treat psoriasis.

## Data Availability Statement

All datasets generated for this study are included in the article/Supplementary Materials.

## Author Contributions

AA-H, RC, and AA-R conceived and designed the study. SH performed all computational studies and analyzed the data. SH and AK wrote the manuscript with inputs and comments from all co-authors. All authors have read and approved the final version of the manuscript.

## Conflict of Interest

The authors declare that the research was conducted in the absence of any commercial or financial relationships that could be construed as a potential conflict of interest.
