# Supplementary material for: Diterpenoids and Triterpenoids From Frankincense Are Excellent Anti-psoriatic Agents: An in silico Approach
Source: Front Chem. 2020 Jun 25;8:486. doi: 10.3389/fchem.2020.00486 (PMC7330179; doi:10.3389/fchem.2020.00486)
Supplement: Supplementary file 1 [file Data_Sheet_1.docx]

**Supporting Information**

**Diterpenoids and Triterpenoids from Frankincense are Excellent Anti-Psoriatic Agents: *In silico* Approach**

**Sobia Ahsan Halim^1^, Ajmal Khan^1^, Rene Csuk^2^, Ahmed Al-Rawahi^1^, Ahmed Al-Harrasi^1^***

^1^Natural and Medical Sciences Research Center, University of Nizwa, Birkat-ul-Mouz 616, Nizwa, Sultanate of Oman

^2^Organic Chemistry, Martin-Luther University Halle-Wittenberg, Kurt-Mothes-Str. 2, D-06120 Halle (Saale), Germany

**Running title: Anti-Psoriatic agents from frankincense**

*Corresponding author

**Prof. Dr. Ahmed Al-Harrasi**

Natural and Medical Sciences Research Center, University of Nizwa, P.O Box 33, Postal Code 616, Birkat Al Mauz, Nizwa, Sultanate of Oman (phone: +96825446328; e-mail: [aharrasi@unizwa.edu.om](mailto:aharrasi@unizwa.edu.om)

**Contents**

- **Table S1. Re-docking and Scoring results of MOE, MVD and ADT vina**
- **Table S2. The Best Predicted Inhibitors by MOE, MVD and ADT Vina**
- **Table S3. MOE Docking Scores of Compounds 1-46 on the selected drug targets**
- **Table S4. MVD Docking Scores of Compounds 1-46 on the selected drug targets**
- **Table S5. ADT Vina Docking Scores of Compounds 1-46 on the selected drug targets**
- **Figure S1. The binding modes of compounds 18, 28, 29 and 32 in the ligand binding site of IL-17. IL-17 dimer (Chain A and B) (PDB Code: 5HI5) is shown in complex with antibody FAB fragments. The compounds are depicted in magenta color and hydrogen bonds are displayed in green lines.**
- **Figure S2. The docked orientations of compounds 20, 36, 38, 40, 43 and 45 at the TNFα receptor binding site. The ligands are shown in purple sticks, H-bonds are demonstrated in green lines and binding residues are presented in cyan stick model.**

**Table S1. Re-docking and Scoring results of MOE, MVD and ADT vina**

| 1. **Used in Re-docking Calculations** | | | | | | | | | |
| --- | --- | --- | --- | --- | --- | --- | --- | --- | --- |
| **Targets** | **MOE** | | | **MVD** | | | **ADT Vina** | | |
|  | **Score** | **Rank** | **RMSD** | **Score** | **Rank** | **RMSD** | **Score** | **Rank** | **RMSD** |
| **JAK1** | -12.07 | 5 | 2.20 | -144.522 | 1 | 0.27 | -9.3 | 3 | 2.4 |
| **JAK2** | -12.65 | 4 | 0.99 | -128.415 | 3 | 0.21 | -9.8 | 3 | 0.4 |
| **JAK3** | -10.38 | 9 | 1.85 | -129.41 | 2 | 0.15 | -8.6 | 3 | 0.96 |
| **iNOS** | -12.75 | 2 | 1.98 | -135.825 | 2 | 0.56 | -9 | 4 | 1.17 |
| **eNOS** | -12.64 | 3 | 1.91 | -169.219 | 2 | 1.25 | -8.8 | 3 | 1.4 |
| **IL-17** | -13.09 | 1 | 2.25 | -197.544 | 1 | 0.25 | -13.4 | 1 | 0.32 |
| **IL-36*γ*** | -15.21 | 1 | 1.68 | -128.481 | 3 | 1.45 | -8.9 | 1 | 0.52 |
| **PPAR-γ** | -15.21 | 1 | 1.15 | -144.91 | 1 | 1.29 | -9.1 | 1 | 1.1 |
| **MAPK2** | -13.54 | 1 | 0.88 | -139.886 | 1 | 0.35 | -9.5 | 1 | 0.23 |
| **TNF-α** | -12.15 | 2 | 2.89 | -177.538 | 1 | 1.2 | -8.9 | 3 | 0.5 |
| **B) Not Included in Re-docking** | | | | | | | | | |
| **IL-1α** | | | | | | | | | |
| IX207-887 | -11.9 | 3 | --- | -131.25 | 5 | --- | -7.55 | 7 | --- |
| RP54745 | -11.87 | 4 | --- | -133.44 | 2 | --- | -8.5 | 2 | --- |
| **IL-1β** |  | | | | | | | | |
| Byakandelicol | -9.95 | 2 | --- | -127.0 | 6 | --- | -7.4 | 3 | --- |
| diacerein | -9.54 | 5 | --- | -131.55 | 2 | --- | -8.4 | 1 | --- |
| **IL-13** | | | | | | | | | |
| Suplatast (Tosilate) | -11.64 | 4 | --- | -122.56 | 2 | --- |  |  | --- |
| **IL-22** | | | | | | | | | |
| GSK2981278 | -10.5 | 3 | --- | -99.86 | 1 | --- | -7.9 | 1 | --- |
| **IL-12/23** | | | | | | | | | |
| Apilimod | -10.7 | 2 | --- | -132.65 | 5 | --- | -8.1 | 7 | --- |
| Isomucronulatol | -10.15 | 8 | --- | -132.44 | 6 | --- | -8.4 | 3 | --- |
| Tyrphostin A1 | -10.22 | 7 | --- | -135.66 | 2 | --- | -8.15 | 7 | --- |
| **IFN-*γ*** | | | | | | | | | |
| AX-024 | -10.1 | 3 | --- | -135.45 | 2 | --- | -8.15 | 7 | --- |
| Pralnacasan | -9.75 | 6 | --- | -132.25 | 4 | --- | -8.35 | 3 | --- |
| **NF-κB** | | | | | | | | | |
| (-)-DHMEQ | -10.9 | 4 | --- | -112.54 | 2 | --- | -8.35 | 3 | --- |
| Daxanabinol | -10.75 | 6 | --- | -110.22 | 3 | --- | -8.15 | 7 | --- |
| **STAT3** | | | | | | | | | |
| STAT3-IN-3 | -10.15 | 1 | --- | -116.45 | 2 | --- | -6.6 | 1 | --- |
| STAT2-IN-1 | -10.11 | 2 | --- | -115.654 | 4 | --- | -6.3 | 3 | --- |

**Table S2. The Best Predicted Inhibitors by MOE, MVD and ADT Vina**

| **S. No.** | **Targets** | **MOE** | **MVD** | **ADT Vina** |
| --- | --- | --- | --- | --- |
|  |  | **Best Predicted Inhibitors** | | |
| **1** | JAK1 | 1-46 | 12, 28-34, 36-37, 40-41, 43-46 | 1-46 |
| **2** | JAK2 | 1-46 | All compounds (2, 19, 26, 31)* | All compounds (32, 39, 43, 45)* |
| **3** | JAK3 | 1-46 | All compounds (1, 4-5, 13, 23, 26-27, 31, 41)* | All compounds (34, 37, 39, 41-42, 45)* |
| **4** | eNOS | 1-46 | 1, 5-7, 10-11, 15-16, 27, 29, 35, 38 | 1-46 |
| **5** | iNOS | 1-46 | 2-3, 8, 12-13, 18, 21, 26, 29, 31-34, 36-38, 40-46, 44 | All compounds (1-2, 5, 7, 10-11, 15)* |
| **6** | IL-17 | 1-8, 10-19, 21-22, 25-46  (9, 20, 23, 24)***** | 1-46 | All compounds (14, 20, 35, 37, 41-42)***** |
| **7** | TNF-α | 1-25, 27-46 (26)* | 1-2, 4, 6, 12-14, 16, 18, 25-29, 30-37, 38, 40-41, 43-46 | 1-29, 31-46 (30)* |
| **8** | MAPK2 | 1-6, 7-11, 13-28, 31, 32 | 1-11, 13-27, 39, 41 | 1, 5, 7, 10-11, 15-16, 20, 23-25 |
| **9** | PPARγ | 1-11, 14-16, 18, 20-27 | All compounds (29, 34, 38, 40)* | 2, 4-6, 9-15, 18-20, 22, 25, 38, 45 |
| **10** | IL-13 | 28-31, 35, 37-39, 42-43, 46 | 4, 9, 12, 19, 26, 29, 33-34, 37-43, 45-46 | 12, 19, 24, 28-46 |
| **11** | IL-23 | 7, 13, 19, 25, 44, 45 | All compounds (1-2, 6-7, 12-14, 18-19, 21)* | 2, 9, 12, 18-19, 21, 26-46 |
| **12** | IL-36γ | 1-27, 30-32, 36, 40, 43, 45-46 | 5-7, 11, 14-16, 20, 22-24 | 3, 4, 6, 13, 20, 22-24 |
| **13** | IFNγ | 5, 10-12, 26, 46 | 1-46 | 1, 3, 12, 16, 20-22, 28-33, 34-46 |

****The rejected compounds are shown in parenthesis***

**Table S3**. **MOE Docking Scores of Compounds 1-46 on the selected drug targets**

| **Comp** | **IFN-*γ*** | **IL-36*γ*** | **IL-23** | **JAK1** | **JAK2** | **JAK3** | **eNOS** | **iNOS** | **MAPK2** | **IL-17** | **PPAR-γ** | **TNF-α** | **IL-13** | **IL-1α** | **IL-1β** | **IL-22** | **NF-κB** | **STAT3** |
| --- | --- | --- | --- | --- | --- | --- | --- | --- | --- | --- | --- | --- | --- | --- | --- | --- | --- | --- |
| **1** | -8.1 | -11.5 | -8.9 | -10.7 | -10.4 | -9.8 | -11.2 | -11.2 | -11.0 | -10.3 | -9.8 | -9.9 | -7.4 | -6.9 | -9.1 | -7.0 | -7.7 | -8.1 |
| **2** | -8.2 | -10.7 | -9.1 | -10.4 | -10.5 | -9.3 | -11.0 | -10.7 | -10.3 | -9.5 | -9.9 | -9.2 | -7.2 | -10.4 | -7.9 | -7.2 | -6.9 | -7.2 |
| **3** | -7.6 | -10.5 | -8.7 | -11.0 | -10.1 | -10.6 | -10.3 | -10.7 | -9.6 | -9.6 | -10.4 | -10.1 | -9.2 | -12.0 | -6.5 | -7.7 | -7.8 | -7.5 |
| **4** | -9.2 | -10.3 | -9.3 | -10.9 | -9.5 | -9.4 | -10.7 | -10.9 | -9.9 | -9.5 | -10.4 | -9.4 | -7.9 | -7.2 | -8.2 | -7.4 | -7.6 | -7.5 |
| **5** | -10.3 | -10.6 | -9.0 | -10.2 | -10.1 | -10.1 | -11.5 | -11.0 | -11.2 | -9.6 | -10.0 | -9.4 | -8.0 | -6.8 | -7.5 | -7.7 | -8.5 | -8.1 |
| **6** | -9.0 | -10.1 | -8.8 | -10.4 | -10.0 | -9.5 | -10.8 | -10.5 | -10.2 | -9.2 | -10.0 | -9.1 | -7.9 | -6.5 | -7.7 | -7.0 | -8.2 | -7.9 |
| **7** | -8.5 | -11.7 | -10.1 | -10.7 | -10.6 | -10.5 | -10.8 | -11.2 | -11.1 | -9.9 | -10.0 | -10.6 | -8.6 | -8.9 | -7.9 | -7.4 | -7.8 | -7.9 |
| **8** | -7.8 | -11.5 | -9.7 | -10.4 | -10.2 | -10.4 | -11.2 | -12.0 | -10.6 | -10.6 | -10.0 | -11.2 | -7.8 | -11.0 | -7.6 | -7.9 | -7.9 | -8.2 |
| **9** | -8.7 | -11.2 | -8.7 | -9.9 | -11.0 | -10.3 | -11.4 | -11.4 | -10.0 | -9.4 | -10.8 | -9.7 | -9.0 | -12.2 | -7.8 | -7.0 | -7.4 | -8.5 |
| **10** | -9.6 | -9.9 | -9.0 | -10.6 | -10.1 | -10.0 | -10.7 | -10.7 | -11.1 | -9.7 | -9.9 | -9.5 | -8.2 | -6.9 | -7.2 | -7.0 | -7.0 | -8.3 |
| **11** | -9.3 | -10.5 | -8.6 | -10.3 | -9.9 | -9.7 | -10.2 | -10.8 | -10.5 | -9.3 | -10.6 | -9.1 | -7.8 | -7.0 | -7.6 | -7.8 | -5.9 | -7.9 |
| **12** | -9.7 | -9.5 | -9.2 | -10.2 | -9.9 | -9.3 | -10.8 | -10.2 | -8.7 | -9.3 | -9.3 | -10.0 | -8.1 | -8.6 | -7.3 | -7.7 | -8.2 | -6.9 |
| **13** | -10.0 | -11.6 | -10.9 | -10.9 | -11.1 | -10.5 | -12.2 | -11.7 | -11.2 | -11.0 | -9.7 | -12.0 | -8.7 | -8.3 | -8.6 | -8.6 | -9.3 | -8.1 |
| **14** | -8.7 | -10.5 | -9.9 | -10.9 | -12.3 | -10.4 | -10.8 | -11.3 | -11.4 | -10.1 | -10.3 | -10.9 | -7.4 | -7.8 | -8.3 | -10.1 | -9.9 | -7.0 |
| **15** | -9.0 | -12.7 | -9.7 | -11.8 | -11.5 | -11.3 | -12.7 | -12.0 | -12.3 | -11.7 | -10.6 | -10.9 | -8.9 | -11.7 | -8.8 | -9.6 | -7.7 | -9.1 |
| **16** | -9.5 | -11.8 | -10.0 | -12.1 | -11.8 | -11.4 | -12.3 | -12.3 | -12.4 | -11.2 | -10.6 | -11.0 | -7.9 | -8.7 | -8.3 | -8.7 | -7.9 | -9.3 |
| **17** | -9.7 | -10.8 | -8.6 | -10.1 | -13.1 | -9.9 | -10.7 | -10.7 | -11.8 | -10.0 | -9.7 | -10.0 | -7.6 | -8.9 | -8.6 | -9.4 | -8.6 | -8.2 |
| **18** | -9.3 | -11.2 | -10.2 | -11.4 | -11.1 | -11.2 | -11.4 | -10.4 | -11.4 | -11.0 | -9.7 | -10.7 | -10.9 | -12.0 | -9.0 | -10.3 | -8.5 | -8.0 |
| **19** | -9.2 | -10.4 | -10.6 | -10.4 | -10.3 | -10.3 | -10.7 | -10.7 | -10.5 | -9.7 | -9.9 | -10.3 | -7.5 | -9.4 | -7.5 | -8.2 | -9.4 | -8.3 |
| **20** | -9.6 | -12.3 | -9.8 | -11.2 | -11.9 | -11.0 | -11.7 | -11.1 | -12.7 | -10.6 | -11.1 | -11.3 | -9.6 | -11.3 | -10.0 | -9.6 | -8.5 | -9.4 |
| **21** | -8.9 | -10.6 | -9.6 | -10.0 | -10.5 | -10.2 | -11.7 | -11.9 | -10.2 | -10.3 | -9.7 | -10.8 | -9.2 | -11.8 | -8.3 | -11.2 | -9.1 | -8.8 |
| **22** | -10.1 | -11.8 | -10.6 | -10.9 | -11.6 | -11.3 | -12.0 | -11.3 | -13.4 | -11.3 | -11.5 | -11.0 | -10.1 | -11.8 | -9.8 | -11.1 | -7.5 | -10.0 |
| **23** | -9.7 | -11.9 | -10.0 | -10.7 | -12.1 | -10.9 | -11.7 | -11.6 | -12.0 | -10.1 | -10.9 | -11.5 | -9.9 | -11.4 | -8.6 | -9.7 | -8.4 | -8.2 |
| **24** | -8.9 | -12.5 | -9.9 | -11.0 | -11.4 | -11.0 | -11.0 | -11.3 | -12.4 | -10.0 | -11.6 | -11.1 | -10.1 | -11.4 | -8.4 | -8.7 | -8.4 | -8.4 |
| **25** | -9.3 | -10.9 | -10.0 | -11.0 | -10.4 | -10.4 | -10.6 | -11.2 | -11.3 | -10.0 | -10.1 | -11.1 | -8.0 | -8.4 | -9.5 | -7.4 | -8.0 | -7.7 |
| **26** | -9.5 | -11.0 | -8.6 | -9.4 | -9.8 | -9.3 | -10.8 | -11.0 | -9.9 | -9.2 | -10.0 | -9.1 | -8.0 | -9.5 | -8.6 | -8.6 | -7.8 | -7.7 |
| **27** | -10.7 | -11.5 | -10.3 | -11.5 | -11.4 | -12.0 | -11.5 | -11.3 | -11.0 | -11.2 | -10.9 | -10.9 | -10.5 | -11.8 | -9.7 | -10.3 | -8.4 | -8.1 |
| **28** | -7.8 | -9.9 | -9.6 | -11.7 | -12.3 | -11.5 | -12.9 | -11.0 | -11.2 | -11.8 | -8.7 | -12.0 | -11.2 | -9.5 | -7.0 | -8.2 | -11.7 | -8.0 |
| **29** | -7.3 | -9.1 | -8.0 | -11.6 | -11.9 | -10.4 | -12.5 | -11.3 | -8.5 | -10.0 | -7.2 | -10.3 | -9.9 | -9.2 | -6.3 | -8.3 | -8.7 | -9.2 |
| **30** | -7.2 | -9.7 | -8.4 | -11.3 | -10.7 | -11.4 | -11.4 | -10.7 | -9.0 | -10.2 | -7.4 | -9.7 | -10.6 | -9.7 | -7.0 | -8.3 | -9.1 | -7.8 |
| **31** | -9.0 | -12.1 | -8.5 | -11.6 | -11.0 | -11.0 | -11.4 | -10.7 | -10.0 | -10.6 | -7.0 | -9.8 | -11.3 | -8.6 | -7.1 | -8.7 | -8.0 | -7.1 |
| **32** | -8.7 | -10.8 | -8.8 | -12.3 | -11.4 | -11.0 | -11.1 | -11.7 | -10.3 | -11.1 | -8.2 | -10.7 | -9.8 | -11.0 | -9.9 | -8.2 | -9.7 | -7.5 |
| **33** | -10.1 | -9.5 | -9.4 | -11.6 | -11.2 | -10.9 | -12.2 | -11.2 | -9.3 | -10.2 | -7.0 | -12.2 | -10.1 | -9.8 | -8.1 | -8.0 | -11.3 | -8.2 |
| **34** | -8.4 | -9.6 | -9.3 | -11.5 | -10.1 | -10.5 | -10.5 | -10.4 | -7.8 | -10.8 | -7.1 | -10.8 | -10.3 | -10.7 | -8.2 | -8.8 | -10.8 | -8.2 |
| **35** | -8.9 | -9.5 | -9.4 | -12.4 | -11.5 | -10.8 | -11.3 | -10.7 | -9.9 | -10.8 | -7.7 | -10.9 | -10.6 | -9.3 | -7.2 | -9.3 | -9.8 | -8.2 |
| **36** | -7.8 | -10.3 | -9.4 | -10.7 | -10.4 | -10.5 | -11.0 | -10.6 | -8.1 | -11.0 | -6.7 | -11.4 | -9.7 | -7.9 | -9.0 | -8.4 | -10.7 | -9.4 |
| **37** | -8.0 | -8.9 | -9.3 | -10.5 | -12.7 | -10.6 | -12.0 | -10.4 | -8.2 | -10.8 | -5.8 | -10.5 | -12.0 | -8.1 | -7.9 | -8.1 | -9.4 | -7.1 |
| **38** | -7.2 | -9.0 | -8.9 | -11.3 | -10.9 | -10.3 | -11.8 | -10.6 | -8.3 | -10.5 | -7.4 | -11.6 | -9.7 | -8.4 | -6.4 | -8.4 | -9.1 | -7.5 |
| **39** | -7.2 | -8.8 | -8.8 | -12.0 | -11.8 | -10.3 | -11.4 | -11.2 | -9.5 | -10.3 | -6.5 | -9.8 | -10.6 | -10.2 | -7.2 | -8.4 | -9.0 | -7.4 |
| **40** | -7.3 | -11.6 | -8.0 | -11.3 | -11.6 | -11.8 | -11.0 | -12.6 | -8.9 | -10.1 | -7.7 | -11.7 | -9.0 | -9.1 | -8.2 | -8.1 | -9.8 | -7.5 |
| **41** | -8.5 | -9.7 | -9.0 | -11.4 | -11.4 | -10.7 | -11.4 | -13.1 | -8.6 | -10.3 | -7.9 | -9.8 | -8.9 | -8.4 | -8.3 | -8.1 | -10.2 | -8.6 |
| **42** | -7.3 | -9.5 | -8.3 | -11.8 | -11.0 | -10.6 | -11.1 | -10.1 | -9.5 | -11.6 | -9.2 | -10.4 | -11.6 | -8.8 | -6.4 | -8.0 | -9.5 | -7.7 |
| **43** | -7.8 | -10.3 | -7.9 | -11.1 | -12.8 | -10.9 | -11.5 | -10.9 | -8.9 | -10.2 | -6.4 | -10.3 | -9.8 | -10.0 | -7.1 | -7.8 | -8.3 | -8.2 |
| **44** | -8.5 | -10.2 | -10.6 | -12.0 | -11.4 | -10.8 | -11.5 | -11.2 | -9.1 | -10.3 | -7.7 | -12.0 | -9.9 | -9.6 | -8.4 | -8.3 | -10.1 | -8.9 |
| **45** | -8.6 | -10.3 | -10.1 | -10.1 | -10.4 | -10.4 | -10.6 | -10.8 | -7.7 | -10.0 | -6.6 | -11.7 | -12.6 | -9.5 | -7.7 | -9.1 | -11.1 | -9.2 |
| **46** | -10.6 | -10.7 | -8.6 | -12.1 | -12.6 | -10.8 | -11.6 | -11.2 | -9.7 | -11.6 | -8.6 | -11.0 | -12.1 | -10.5 | -8.3 | -8.4 | -9.6 | -8.6 |

**Table S4**. **MVD Docking Scores of Compounds 1-46 on the selected drug targets**

| **Compounds** | **IFN-*γ*** | **IL-36*γ*** | **IL-23** | **JAK1** | **JAK2** | **JAK3** | **eNOS** | **iNOS** | **MAPK2** | **IL-17** | **PPAR-γ** | **TNF-α** | **IL-13** | **IL-1α** | **IL-1β** | **IL-22** | **NF-κB** | **STAT3** |
| --- | --- | --- | --- | --- | --- | --- | --- | --- | --- | --- | --- | --- | --- | --- | --- | --- | --- | --- |
| **1** | -116.4 | -104.2 | -99.3 | -92.6 | -107.3 | -102.1 | -103.2 | -105.4 | -118.0 | -109.5 | -123.0 | -108.0 | -100.3 | -102.5 | -114.1 | -75.2 | -92.3 | -100.9 |
| **2** | -121.7 | -102.6 | -106.3 | -102.4 | -101.9 | -110.0 | -104.0 | -118.1 | -123.9 | -118.8 | -122.6 | -113.8 | -104.9 | -105.7 | -113.6 | -90.2 | -101.4 | -109.5 |
| **3** | -124.7 | -112.6 | -123.5 | -110.3 | -123.5 | -120.8 | -106.6 | -122.5 | -125.7 | -133.2 | -126.6 | -115.0 | -112.0 | -110.4 | -123.3 | -82.4 | -101.2 | -116.3 |
| **4** | -117.8 | -102.1 | -113.4 | -90.9 | -106.2 | -104.8 | -97.1 | -101.9 | -111.0 | -108.5 | -121.2 | -114.0 | -107.8 | -104.4 | -119.8 | -95.4 | -94.6 | -97.3 |
| **5** | -119.6 | -108.4 | -131.4 | -89.2 | -111.5 | -103.6 | -104.2 | -98.8 | -122.1 | -109.5 | -124.0 | -101.2 | -94.8 | -105.4 | -106.9 | -77.2 | -96.8 | -95.7 |
| **6** | -117.1 | -110.2 | -105.6 | -101.7 | -111.9 | -113.4 | -105.4 | -105.3 | -121.0 | -114.1 | -126.9 | -111.1 | -105.4 | -109.0 | -114.7 | -82.9 | -100.5 | -101.2 |
| **7** | -126.3 | -129.9 | -108.2 | -104.1 | -121.7 | -123.8 | -111.5 | -112.3 | -135.4 | -130.6 | -131.8 | -113.4 | -108.9 | -113.5 | -134.0 | -88.0 | -97.8 | -113.7 |
| **8** | -122.7 | -117.6 | -120.8 | -115.8 | -125.6 | -136.1 | -111.6 | -124.0 | -134.4 | -126.7 | -137.2 | -118.1 | -109.9 | -116.3 | -126.9 | -96.7 | -100.6 | -114.7 |
| **9** | -125.4 | -105.3 | -112.4 | -93.7 | -115.0 | -108.7 | -94.2 | -101.4 | -120.6 | -112.6 | -128.4 | -104.3 | -111.4 | -104.6 | -119.8 | -79.6 | -98.4 | -102.3 |
| **10** | -115.9 | -106.4 | -110.4 | -90.2 | -108.5 | -108.0 | -102.0 | -100.6 | -125.0 | -111.0 | -131.1 | -102.0 | -96.0 | -104.7 | -111.9 | -80.0 | -93.4 | -99.4 |
| **11** | -119.5 | -109.7 | -120.6 | -94.6 | -109.7 | -108.4 | -105.8 | -97.3 | -125.0 | -112.4 | -123.2 | -106.1 | -94.0 | -105.6 | -109.7 | -80.3 | -98.5 | -97.0 |
| **12** | -121.2 | -96.0 | -103.7 | -109.9 | -108.3 | -107.9 | -97.2 | -115.7 | -104.9 | -114.7 | -116.6 | -114.0 | -113.7 | -108.1 | -124.2 | -90.3 | -102.8 | -98.8 |
| **13** | -120.3 | -106.8 | -101.5 | -100.1 | -110.1 | -103.1 | -98.0 | -114.8 | -126.8 | -113.5 | -120.3 | -109.7 | -102.3 | -101.9 | -118.8 | -84.3 | -95.0 | -105.2 |
| **14** | -132.8 | -125.1 | -112.2 | -110.3 | -125.2 | -117.5 | -98.7 | -113.7 | -136.4 | -126.6 | -133.0 | -120.2 | -108.2 | -132.2 | -127.2 | -80.6 | -102.5 | -106.8 |
| **15** | -121.5 | -119.7 | -124.5 | -97.3 | -113.6 | -110.7 | -109.9 | -104.9 | -129.4 | -119.3 | -126.8 | -111.2 | -101.2 | -116.8 | -113.0 | -99.5 | -92.3 | -101.3 |
| **16** | -131.0 | -129.5 | -117.3 | -114.3 | -122.3 | -120.4 | -120.6 | -116.6 | -139.2 | -124.5 | -132.1 | -121.1 | -109.6 | -122.0 | -117.9 | -83.4 | -94.7 | -102.6 |
| **17** | -123.3 | -109.4 | -113.8 | -106.6 | -118.7 | -112.6 | -97.3 | -111.9 | -133.0 | -124.0 | -119.8 | -113.6 | -102.7 | -109.8 | -126.6 | -87.8 | -102.0 | -116.1 |
| **18** | -123.2 | -106.2 | -109.2 | -101.7 | -116.6 | -114.3 | -107.1 | -120.4 | -125.1 | -124.4 | -132.3 | -114.1 | -109.8 | -106.0 | -117.2 | -87.7 | -96.3 | -107.4 |
| **19** | -115.5 | -99.5 | -101.1 | -102.9 | -102.5 | -103.6 | -89.8 | -103.9 | -117.3 | -112.0 | -114.4 | -104.2 | -107.1 | -96.8 | -112.0 | -79.7 | -94.0 | -109.4 |
| **20** | -132.3 | -122.6 | -134.1 | -101.7 | -129.3 | -122.3 | -108.4 | -112.2 | -133.0 | -120.6 | -126.9 | -115.9 | -110.0 | -114.3 | -130.2 | -95.9 | -99.2 | -108.0 |
| **21** | -116.9 | -100.6 | -103.5 | -107.4 | -114.8 | -111.5 | -95.5 | -121.2 | -113.2 | -116.6 | -115.3 | -102.0 | -97.4 | -100.1 | -112.9 | -86.6 | -107.0 | -99.4 |
| **22** | -121.5 | -118.5 | -129.5 | -94.4 | -118.6 | -112.6 | -96.8 | -101.5 | -126.9 | -111.5 | -120.9 | -99.7 | -105.6 | -106.3 | -113.8 | -86.4 | -101.3 | -100.1 |
| **23** | -122.4 | -118.7 | -110.5 | -98.8 | -112.3 | -103.9 | -90.7 | -101.6 | -122.3 | -114.3 | -126.4 | -105.8 | -100.8 | -107.0 | -127.3 | -77.9 | -94.8 | -95.9 |
| **24** | -121.0 | -122.3 | -131.1 | -98.8 | -112.2 | -109.8 | -95.6 | -101.6 | -122.4 | -114.3 | -123.4 | -102.2 | -100.8 | -104.4 | -118.5 | -77.9 | -98.0 | -93.7 |
| **25** | -124.7 | -102.9 | -116.6 | -101.0 | -118.9 | -110.6 | -95.4 | -108.5 | -125.1 | -119.7 | -122.1 | -115.1 | -105.1 | -105.9 | -125.9 | -82.9 | -101.5 | -102.3 |
| **26** | -131.4 | -103.8 | -110.9 | -105.5 | -106.6 | -108.0 | -100.4 | -121.5 | -116.8 | -115.9 | -114.4 | -119.8 | -116.2 | -104.8 | -113.0 | -80.6 | -102.5 | -108.5 |
| **27** | -117.9 | -102.3 | -104.6 | -99.9 | -109.9 | -100.5 | -102.3 | -104.7 | -115.7 | -113.4 | -105.6 | -105.1 | -104.4 | -102.1 | -110.5 | -78.7 | -86.6 | -103.1 |
| **28** | -101.6 | -91.8 | -103.6 | -101.4 | -118.8 | -105.4 | -78.9 | -96.4 | -92.7 | -106.0 | -100.0 | -105.8 | -96.9 | -96.9 | -81.6 | -75.9 | -85.9 | -96.2 |
| **29** | -114.4 | -78.2 | -118.8 | -107.8 | -114.3 | -105.8 | -103.3 | -105.9 | -97.0 | -114.5 | -99.2 | -105.8 | -105.8 | -93.1 | -89.9 | -83.1 | -97.5 | -95.4 |
| **30** | -117.0 | -82.0 | -119.7 | -119.6 | -119.6 | -110.5 | -91.2 | -105.7 | -101.1 | -115.6 | -111.6 | -111.9 | -102.0 | -101.8 | -93.5 | -82.8 | -94.8 | -98.3 |
| **31** | -123.0 | -83.4 | -136.0 | -116.9 | -98.5 | -108.7 | -100.6 | -130.7 | -110.1 | -132.2 | -111.6 | -112.9 | -109.7 | -111.7 | -97.9 | -96.8 | -91.8 | -116.8 |
| **32** | -103.3 | -86.7 | -113.9 | -120.1 | -105.8 | -102.5 | -83.1 | -125.1 | -99.2 | -107.2 | -101.7 | -106.5 | -97.0 | -97.6 | -81.3 | -83.9 | -86.9 | -98.1 |
| **33** | -108.6 | -78.7 | -112.9 | -108.7 | -118.8 | -106.9 | -90.8 | -109.0 | -96.8 | -115.5 | -105.9 | -104.6 | -105.1 | -97.5 | -74.7 | -80.5 | -100.6 | -102.0 |
| **34** | -107.2 | -83.0 | -118.0 | -117.9 | -115.8 | -114.9 | -93.6 | -111.8 | -87.8 | -119.2 | -101.7 | -120.8 | -111.7 | -105.2 | -111.9 | -85.6 | -105.7 | -100.5 |
| **35** | -111.3 | -81.0 | -112.4 | -99.4 | -119.1 | -101.1 | -82.8 | -99.7 | -97.4 | -108.1 | -102.3 | -99.2 | -96.4 | -104.8 | -105.9 | -81.4 | -95.0 | -91.1 |
| **36** | -121.7 | -92.4 | -131.2 | -116.5 | -109.7 | -127.2 | -95.6 | -139.1 | -109.1 | -116.2 | -112.8 | -122.6 | -110.5 | -103.3 | -104.5 | -82.7 | -94.4 | -103.5 |
| **37** | -122.3 | -96.2 | -133.6 | -126.5 | -133.8 | -128.9 | -104.2 | -124.1 | -78.7 | -125.3 | -120.7 | -131.8 | -124.0 | -116.2 | -114.6 | -88.5 | -113.4 | -101.1 |
| **38** | -113.9 | -70.8 | -110.4 | -112.1 | -99.3 | -101.9 | -97.1 | -105.2 | -88.1 | -111.0 | -94.6 | -110.7 | -99.7 | -83.6 | -84.6 | -71.0 | -87.4 | -97.6 |
| **39** | -136.7 | -84.5 | -128.1 | -100.4 | -108.0 | -124.7 | -94.6 | -106.1 | -102.8 | -117.1 | -115.4 | -102.4 | -107.1 | -94.1 | -91.4 | -88.5 | -92.5 | -100.0 |
| **40** | -109.0 | -87.3 | -119.0 | -107.7 | -114.7 | -103.5 | -86.0 | -107.6 | -87.9 | -119.1 | -97.0 | -114.0 | -104.1 | -102.3 | -102.1 | -77.7 | -89.5 | -91.1 |
| **41** | -109.3 | -84.8 | -107.4 | -121.0 | -105.4 | -99.7 | -94.2 | -112.5 | -106.4 | -109.6 | -105.3 | -105.1 | -116.6 | -98.1 | -95.4 | -75.6 | -100.0 | -100.3 |
| **42** | -109.7 | -78.6 | -134.1 | -104.0 | -116.7 | -115.3 | -88.4 | -110.9 | -99.5 | -119.0 | -109.2 | -102.2 | -107.8 | -100.6 | -118.1 | -77.9 | -112.4 | -99.6 |
| **43** | -116.1 | -79.1 | -118.4 | -122.1 | -119.7 | -115.0 | -103.1 | -118.4 | -80.8 | -129.2 | -119.4 | -117.7 | -115.9 | -98.4 | -117.0 | -91.1 | -95.3 | -104.1 |
| **44** | -97.3 | -84.9 | -100.2 | -98.3 | -109.1 | -101.2 | -73.7 | -127.9 | -92.8 | -100.8 | -96.8 | -94.3 | -89.7 | -89.3 | -78.8 | -74.1 | -82.4 | -83.4 |
| **45** | -94.3 | -69.0 | -109.5 | -101.7 | -113.2 | -108.0 | -78.7 | -107.4 | -70.8 | -106.4 | -102.7 | -103.6 | -103.8 | -87.8 | -103.4 | -88.2 | -90.2 | -82.9 |
| **46** | -96.2 | -79.8 | -100.6 | -110.7 | -103.7 | -92.9 | -74.7 | -120.7 | -95.5 | -99.4 | -94.1 | -98.8 | -97.3 | -87.1 | -76.6 | -78.1 | -92.9 | -92.1 |

**Table S5**. **ADT Vina Docking Scores of Compounds 1-46 on the selected drug targets**

| **Compounds** | **IFN-*γ*** | **IL-36*γ*** | **IL-23** | **JAK1** | **JAK2** | **JAK3** | **eNOS** | **iNOS** | **MAPK2** | **IL-17** | **PPAR-γ** | **TNF-α** | **IL-13** | **IL-1α** | **IL-1β** | **IL-22** | **NF-κB** | **STAT3** |
| --- | --- | --- | --- | --- | --- | --- | --- | --- | --- | --- | --- | --- | --- | --- | --- | --- | --- | --- |
| **1** | -6.8 | -6.5 | -6.1 | -7.5 | -8.4 | -7.9 | -8 | -6.4 | -7.7 | -7.5 | -6.7 | -7.3 | -6.5 | -5.9 | -5.2 | -5.4 | -5.5 | -4.7 |
| **2** | -6.3 | -4.9 | -6.8 | -7.9 | -8.1 | -7.6 | -6.5 | -6.3 | -5.9 | -7.6 | -6.8 | -7.6 | -6.1 | -6.1 | -5.6 | -5.9 | -5.7 | -4.8 |
| **3** | -6.8 | -6.9 | -6.1 | -7.3 | -9.4 | -8.3 | -7.1 | -6.7 | -5.8 | -8 | -6.7 | -7.3 | -6.7 | -5.6 | -5.8 | -4.6 | -5.5 | -4.7 |
| **4** | -6.3 | -7.5 | -6.8 | -8.3 | -8.1 | -7.7 | -7.4 | -6.9 | -5.9 | -6.7 | -7.1 | -7.3 | -6.7 | -5.9 | -5.2 | -5.7 | -5.6 | -5.5 |
| **5** | -6.2 | -7.2 | -6.6 | -8.9 | -9.6 | -8.6 | -7.1 | -6.3 | -9.2 | -8.2 | -7.9 | -8.9 | -6.3 | -5.7 | -5.5 | -4.7 | -5.4 | -4.9 |
| **6** | -6.1 | -7.1 | -6.4 | -7.4 | -9 | -8.6 | -6.8 | -6.7 | -6.3 | -8.1 | -6.9 | -7.5 | -6.4 | -5.6 | -5.1 | -6.4 | -5.3 | -4.7 |
| **7** | -6.3 | -6.4 | -6.2 | -8.2 | -8.6 | -7.8 | -6.9 | -6.3 | -7 | -7.4 | -6.5 | -7.3 | -6.5 | -5.9 | -5.4 | -4.9 | -5.8 | -5 |
| **8** | -6.7 | -6.3 | -5.9 | -8.4 | -8.8 | -8.3 | -7.2 | -6.9 | -6.2 | -8.5 | -6.6 | -6.8 | -6.6 | -6.1 | -5.1 | -4.7 | -5.6 | -5.2 |
| **9** | -6.4 | -5.8 | -6.9 | -7.8 | -8.3 | -8.5 | -7.2 | -6.8 | -6.3 | -7.6 | -6.9 | -7.3 | -6.5 | -5.9 | -5.4 | -6.5 | -5.7 | -5.1 |
| **10** | -6.5 | -6.7 | -6.6 | -9.1 | -9.4 | -8.3 | -7.6 | -6.3 | -8.9 | -7.7 | -7.4 | -8.1 | -6.3 | -5.9 | -5.4 | -5.8 | -5.4 | -4.9 |
| **11** | -6.2 | -6.5 | -6 | -8.4 | -8.9 | -8.5 | -7 | -6.4 | -9.2 | -7.6 | -7.6 | -8.3 | -6.2 | -5.3 | -5.4 | -5.1 | -5.4 | -4.7 |
| **12** | -6.3 | -5.4 | -6.7 | -6 | -7.1 | -6.7 | -7.4 | -6.5 | -3.6 | -6.9 | -6.7 | -7.5 | -6.5 | -5.7 | -5.1 | -4.4 | -5.7 | -4.7 |
| **13** | -6.6 | -7.4 | -6.9 | -8.4 | -8.6 | -8.3 | -7.3 | -6.9 | -6.9 | -7.3 | -7.8 | -7.9 | -6.4 | -6.1 | -5.6 | -6.2 | -5.9 | -5.4 |
| **14** | -6.8 | -7 | -6.7 | -8 | -9.1 | -7.9 | -7.4 | -7 | -6.9 | -6.8 | -7.4 | -8.1 | -6.5 | -6.8 | -5.5 | -5.8 | -5.6 | -5.4 |
| **15** | -7 | -7.1 | -6.4 | -9 | -9 | -8.2 | -7.2 | -6.8 | -8.3 | -7.9 | -7.6 | -7.7 | -7 | -5.7 | -5.3 | -6 | -5.8 | -5.1 |
| **16** | -7.3 | -6.9 | -6.6 | -8.1 | -9.4 | -8.6 | -8 | -7.3 | -7.5 | -6.8 | -7 | -7.3 | -6.8 | -5.7 | -5.5 | -4.8 | -5.8 | -5.1 |
| **17** | -6.6 | -5.2 | -6.6 | -8.4 | -8.4 | -8.2 | -7.5 | -6.8 | -5.4 | -8 | -6.5 | -7.6 | -6.7 | -6 | -5.8 | -5.2 | -6 | -5.2 |
| **18** | -6.8 | -6.2 | -6.7 | -7.9 | -7.9 | -8.4 | -7.5 | -7.6 | -5.4 | -7.2 | -7.5 | -6.9 | -6.5 | -6.1 | -5.8 | -5.7 | -5.7 | -5.4 |
| **19** | -6.5 | -5.4 | -7.2 | -7.9 | -8.2 | -8.1 | -7.2 | -6.7 | -5.8 | -7.8 | -6.9 | -7.6 | -7.1 | -5.9 | -5.7 | -5.6 | -6.3 | -5.2 |
| **20** | -7.5 | -7.4 | -7 | -8.4 | -9 | -8.1 | -7.7 | -7.1 | -7.2 | -6.8 | -7.5 | -7.7 | -6.7 | -6.2 | -6 | -5.4 | -6.1 | -4.9 |
| **21** | -6.7 | -5.3 | -6.3 | -6.7 | -7.8 | -7 | -7 | -6.7 | -4.4 | -7 | -6.1 | -6.5 | -6.1 | -5.7 | -5.5 | -5.4 | -5.8 | -4.7 |
| **22** | -7.9 | -7.5 | -6.8 | -8.6 | -9 | -8.1 | -7.5 | -7.5 | -6.9 | -7 | -7.5 | -7.2 | -7 | -6.1 | -6 | -5.3 | -6.4 | -5.3 |
| **23** | -7.2 | -7.5 | -6.7 | -8 | -9.1 | -8.6 | -7.4 | -7.6 | -7.4 | -8.2 | -7.2 | -7.4 | -7.1 | -6.4 | -5.8 | -4.9 | -6.1 | -5 |
| **24** | -7.1 | -7.5 | -6.8 | -8 | -9.1 | -8.6 | -7.4 | -7.6 | -7.4 | -8.2 | -7.2 | -7.4 | -7.3 | -6.4 | -5.8 | -4.9 | -6.1 | -5 |
| **25** | -6.1 | -5.5 | -6.3 | -7.4 | -8.2 | -7.6 | -7.3 | -6.5 | -6.9 | -7.6 | -6.8 | -7.5 | -6.5 | -5.7 | -5.1 | -4.7 | -5.6 | -5 |
| **26** | -6.5 | -5.3 | -7 | -8.1 | -8.4 | -8.4 | -6.8 | -7 | -5.4 | -8.8 | -6.7 | -8.4 | -6.8 | -5.6 | -5.9 | -6.6 | -5.8 | -5.1 |
| **27** | -6.7 | -5.7 | -7.1 | -8.4 | -8.9 | -7.9 | -7.2 | -7.5 | -4.8 | -7.5 | -6.8 | -7.1 | -6.6 | -6 | -6.3 | -6.1 | -5.9 | -5.7 |
| **28** | -7.7 | -5.8 | -8.2 | -9 | -10.4 | -8.2 | -8.8 | -8.8 | -7.7 | -8.4 | -6.7 | -7.9 | -8.6 | -7.8 | -7.3 | -5.9 | -6.9 | -6 |
| **29** | -7.7 | -5.5 | -8 | -7.8 | -8.2 | -7 | -8.2 | -8.7 | -6 | -7.5 | -6.7 | -7.2 | -8.7 | -7.9 | -7.2 | -6 | -7.2 | -5.8 |
| **30** | -7.9 | -5.6 | -7.8 | -9.1 | -8.9 | -7.8 | -8.1 | -9 | -6.1 | -7.4 | -6.7 | -6.5 | -8.4 | -6.6 | -6.4 | -5.9 | -6.8 | -5.7 |
| **31** | -8.4 | -5.7 | -8 | -9 | -7.9 | -8.3 | -8.1 | -8.1 | -5.5 | -7.6 | -6.9 | -7.8 | -9.2 | -8.9 | -6.4 | -5.9 | -7.7 | -5.9 |
| **32** | -8.3 | -5.7 | -7.7 | -9.6 | -7.4 | -8.4 | -8.9 | -8 | -6.1 | -8.8 | -7.1 | -7.7 | -8.6 | -7.6 | -6.7 | -6.1 | -7.2 | -6 |
| **33** | -7.9 | -5.6 | -8.2 | -7.3 | -8.7 | -9 | -8.1 | -8.8 | -6.1 | -7.6 | -7.1 | -7.5 | -8.3 | -7 | -6.3 | -5.9 | -7.8 | -5.6 |
| **34** | -8.2 | -5 | -7.8 | -7.7 | -7.2 | -6.2 | -8.4 | -8.7 | -6.2 | -7.5 | -6.8 | -7.4 | -8.1 | -7.2 | -6.8 | -5.9 | -7.1 | -6 |
| **35** | -8 | -5.3 | -7.6 | -8 | -8.1 | -8.2 | -8.2 | -8.9 | -6.2 | -7.1 | -6.5 | -8.8 | -8.1 | -8 | -5.9 | -6.1 | -6.9 | -5.9 |
| **36** | -7.8 | -5.2 | -7.5 | -7.7 | -7.2 | -8.8 | -8 | -9.1 | -6.6 | -7.3 | -6.9 | -8.8 | -7.8 | -7.1 | -6.9 | -5.6 | -6.7 | -5.8 |
| **37** | -7.7 | -5.5 | -7.4 | -7.6 | -7.2 | -6.6 | -8.3 | -8.8 | -5.58 | -6.3 | -7.1 | -8 | -7.8 | -7 | -6.8 | -6.1 | -6.9 | -5.5 |
| **38** | -7.8 | -5.3 | -7.7 | -8.5 | -8 | -7.8 | -8.9 | -7.8 | -6 | -8.1 | -7.7 | -9.8 | -8.7 | -6.8 | -6.9 | -6.9 | -7 | -5.6 |
| **39** | -8.2 | -5.4 | -7.9 | -9 | -6.6 | -6.8 | -8 | -8.7 | -6.2 | -9.4 | -7.2 | -9 | -8.6 | -6.9 | -6.3 | -6 | -7 | -5.7 |
| **40** | -8.1 | -5.7 | -7.9 | -7.7 | -7.6 | -7.9 | -8 | -8.9 | -5.8 | -7.7 | -6.6 | -9 | -8.1 | -7.8 | -7.7 | -6.7 | -7.2 | -5.7 |
| **41** | -7.4 | -5.5 | -8.6 | -7.6 | -7.2 | -6.7 | -8.1 | -8 | -6.5 | -6.8 | -7.2 | -8.7 | -7.9 | -7.4 | -6 | -6.3 | -7.3 | -5.9 |
| **42** | -8.1 | -6.3 | -7.8 | -8.4 | -8.3 | -7.1 | -8.2 | -9.1 | -6.8 | -6.9 | -6.2 | -8.8 | -8.2 | -6.9 | -5.7 | -5.7 | -7.5 | -5.8 |
| **43** | -7.4 | -5.6 | -7.7 | -8.3 | -6.4 | -8.4 | -7.9 | -8.9 | -6.1 | -7.4 | -7 | -8.8 | -8.1 | -7 | -5.8 | -5.9 | -7 | -5.7 |
| **44** | -7.9 | -5.8 | -8.6 | -8.4 | -9.9 | -8.3 | -8.3 | -8.5 | -6.1 | -8.3 | -7.4 | -8.6 | -8.7 | -7.5 | -7.5 | -5.6 | -7.1 | -5.8 |
| **45** | -8.5 | -5.8 | -8.6 | -7.7 | -6.9 | -7 | -8.1 | -9.2 | -6.3 | -7.7 | -8.3 | -8.8 | -8.4 | -7.2 | -5.9 | -7.5 | -7.1 | -6.4 |
| **46** | -8.2 | -5.6 | -8.3 | -9.4 | -7.7 | -8.3 | -8.5 | -8.2 | -5.9 | -8 | -6.6 | -8.9 | -8.4 | -7.2 | -6.7 | -6.2 | -7.3 | -5.9 |


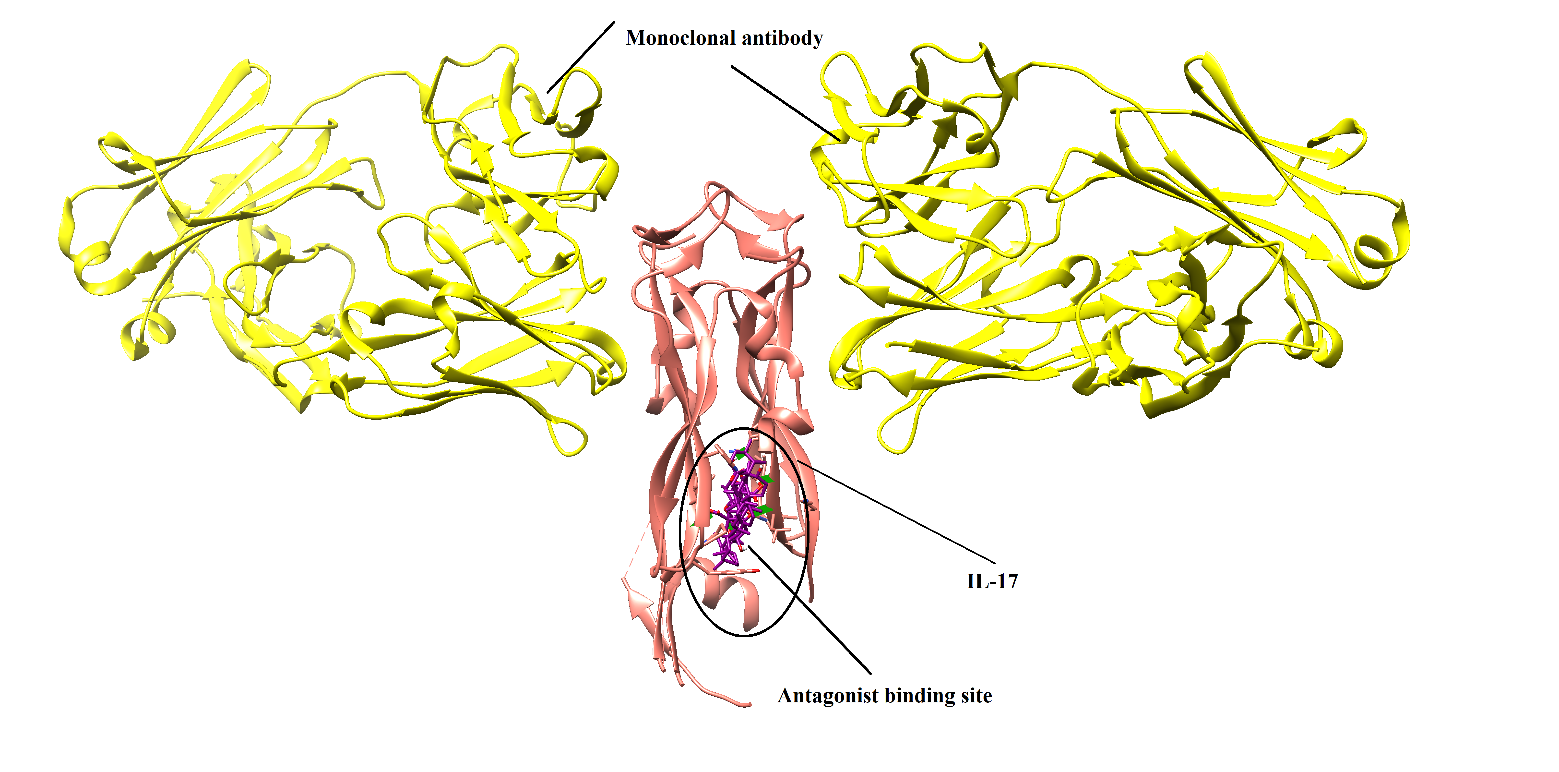


(a)


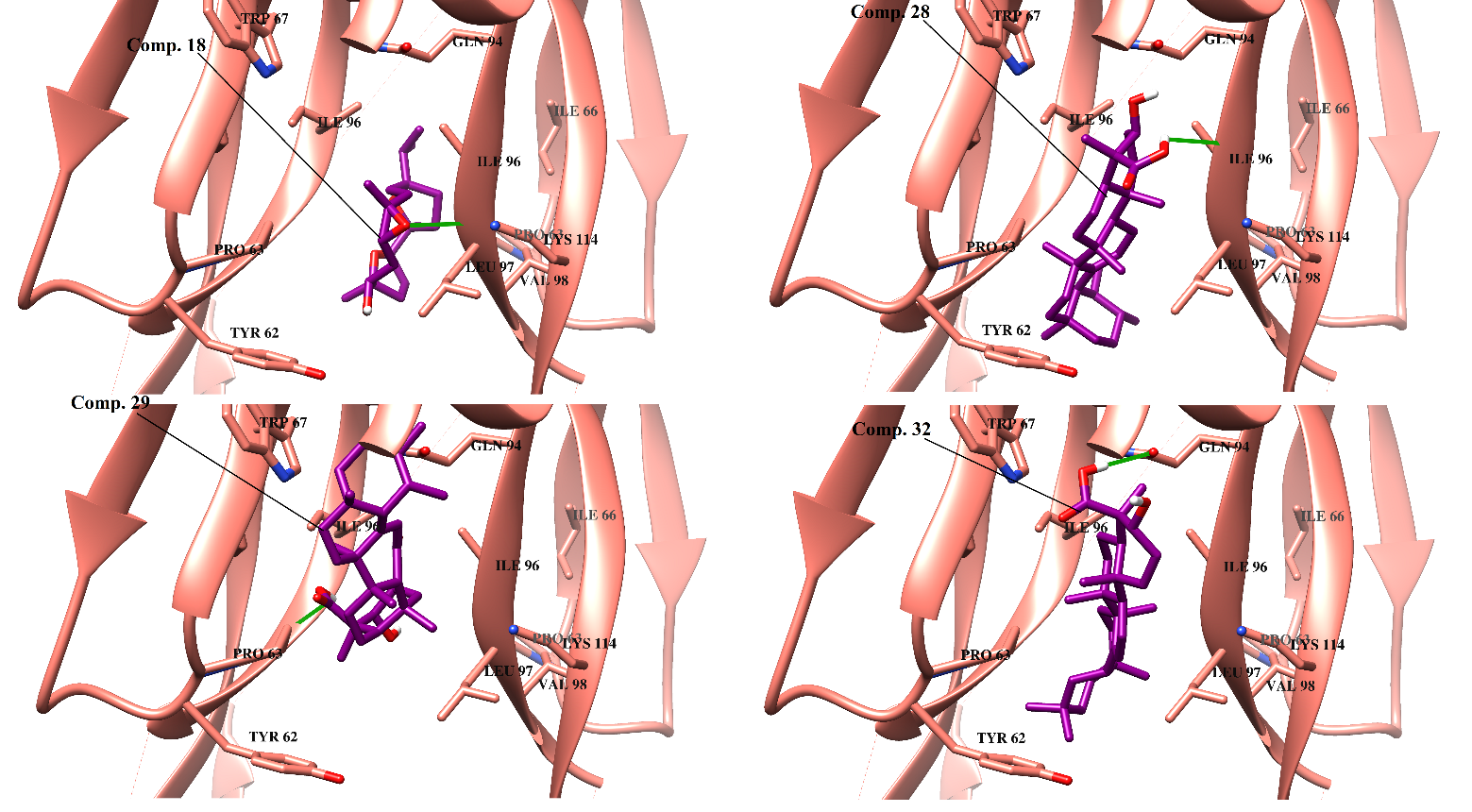


(b)

Figure S1. The binding modes of compounds **18, 28, 29** and **32** in the ligand binding site of IL-17. IL-17 dimer (Chain A and B) (PDB Code: 5HI5) is shown in complex with antibody FAB fragments. The compounds are depicted in magenta color and hydrogen bonds are displayed in green lines.


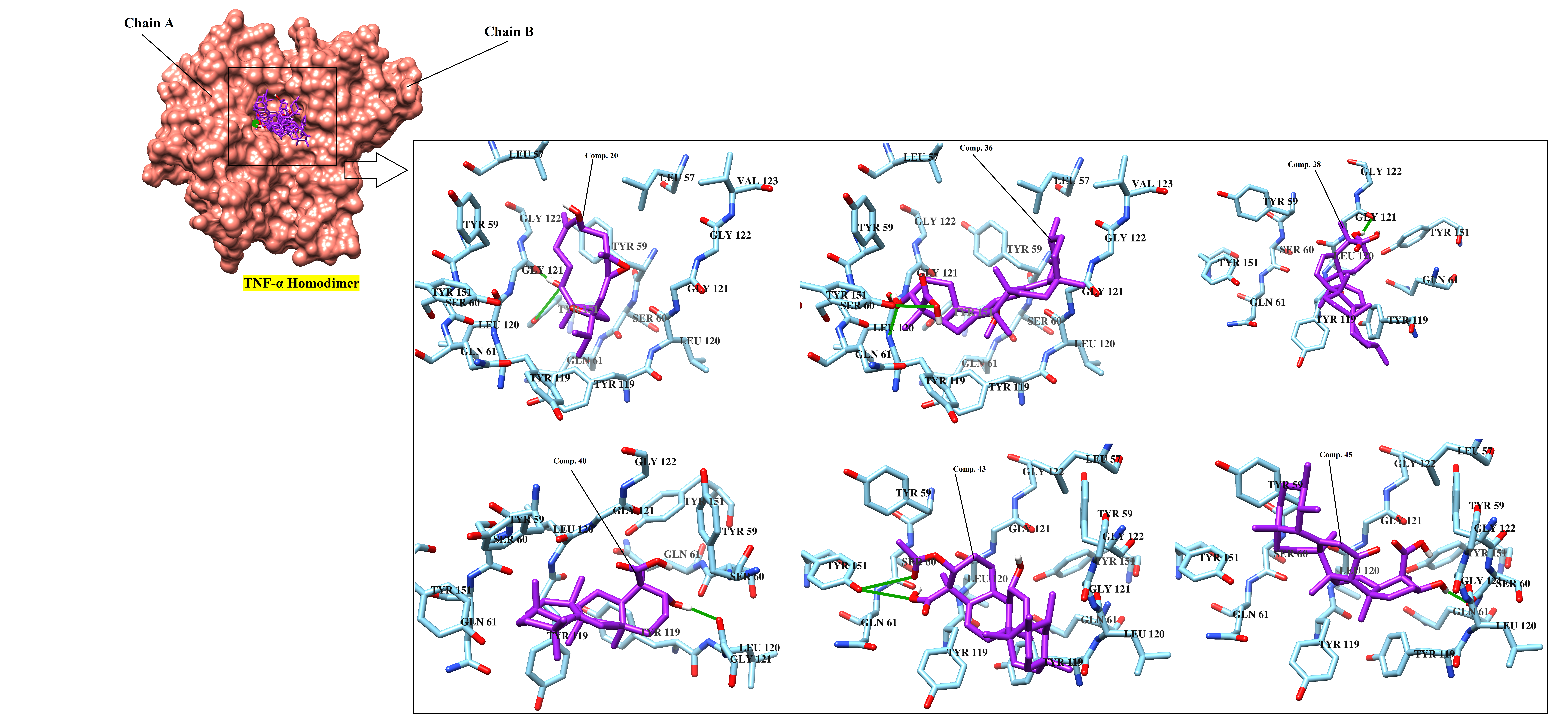


Figure S2. The docked orientations of compounds **20, 36, 38, 40, 43** and **45** at the TNFα receptor binding site. The ligands are shown in purple sticks, H-bonds are demonstrated in green lines and binding residues are presented in cyan stick model.
